# “Abraxane‐Like” Radiosensitizer for In Situ Oral Cancer Therapy

**DOI:** 10.1002/advs.202309569

**Published:** 2024-07-07

**Authors:** Zijian Gong, Yixuan Fu, Yuan Gao, Fei Jiao, Qinzhi Su, Xiao Sang, Binglin Chen, Xuliang Deng, Xinyu Liu

**Affiliations:** ^1^ Central Laboratory Department of Geriatric Dentistry Beijing Laboratory of Biomedical Materials NMPA Key Laboratory for Dental Materials National Engineering Laboratory for Digital and Material Technology of Stomatology Peking University School and Hospital of Stomatology Beijing 100081 P. R. China; ^2^ Biomedical Engineering Department Peking University Beijing 100191 P. R. China

**Keywords:** biomineralization, calcium peroxide, HSA, oral cancer, radiosensitizer

## Abstract

Radiotherapy plays a vital role in cancer therapy. However, the hypoxic microenvironment of tumors greatly limits the effectiveness, thus it is crucial to develop a simple, efficient, and safe radiosensitizer to reverse hypoxia and ameliorate the efficacy of radiotherapy. Inspired by the structure of canonical nanodrug Abraxane, herein, a native HSA‐modified CaO_2_ nanoparticle system (CaO_2_‐HSA) prepared by biomineralization‐induced self‐assembly is developed. CaO_2_‐HSA will accumulate in tumor tissue and decompose to produce oxygen, altering the hypoxic condition inside the tumor. Simultaneously, ROS and calcium ions will lead to calcium overload and further trigger immunogenic cell death. Notably, its sensitizing enhancement ratio (SER = 3.47) is much higher than that of sodium glycididazole used in the clinic. Furthermore, in animal models of in situ oral cancer, CaO_2_‐HSA can effectively inhibit tumor growth. With its high efficacy, facile preparation, and heavy‐metal free biosafety, the CaO_2_‐HSA‐based radiosensitizer holds enormous potential for oral cancer therapy.

## Graphical abstract

Radiotherapy's effectiveness in cancer treatment is hindered by tumor hypoxia. To address this, an “Abraxane‐like” radiosensitizer is developed CaO_2_‐HSA prepared by biomineralization‐induced self‐assembly strategy. It accumulates in tumors, producing oxygen, inducing calcium overload, and triggering immunogenic cell death. With a sensitizing enhancement ratio of 3.47, CaO_2_‐HSA shows superior efficacy and potential for oral cancer therapy.

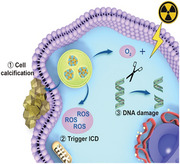

## 1 Introduction

Oral squamous cell carcinoma (OSCC), the most common malignant tumor in the oral cavity, frequently has a harsh impact on patients' vocalization, breathing, chewing, swallowing, and other vital physiological functions, and even threatens their lives due to its poor five‐year survival rate (<60%).^[^ ^1^ ^]^ Currently, surgery and radiotherapy still remain the standard treatment options.^[^ ^2^ ^]^ Compared with surgical treatment that carries the risk of postoperative impairment, radiotherapy has superior potential in treating OSCC owing to its non‐invasiveness and well tolerability. However, the hypoxic microenvironment in large solid tumors would alleviate the DNA damage effect of ionizing radiation in tumor cells, resulting in unsatisfactory radiotherapy outcomes. Gray et. al. reported that the radiation exposure required to kill hypoxic cells is three times that of normal cells,^[^ ^3^ ^]^ and simply increasing the radiation dose would otherwise lead to unfavorable side effects. Therefore, remodeling the hypoxic microenvironment has become a promising strategy to ameliorate the efficacy of radiotherapy.

Radiosensitizers that can mimic oxygen to prevent DNA repair after radiation are currently being used in clinics, whose purpose is to increase the sensitivity of tumors to ionizing radiation and to promote tumor inactivation.^[^ ^4^ ^]^ For instance, electron‐affinic compounds like nimorazole^[^ ^5^ ^]^ and sodium glycididazole^[^ ^6^ ^]^ have been employed for the treatment of neck and head cancer in Denmark and China, respectively. However, as chemical small molecules, these nitroimidazole derivatives‐based radiosensitizers suffer from their potential neurotoxicity, rapid clearance leading to poor bioavailability, and limited targeting function resulting in low tumor accumulation.

The advances of nanomedicine open up opportunities for developing novel radiosensitizers. Calcium peroxide (CaO_2_), as a common non‐toxic oxygen supply agent, has been widely applied to not only aquaculture and sewage treatment but also tumor therapy, especially the nanoscale CaO_2_.^[^ ^7^ ^]^ Recently, CaO_2_ nanoparticles have been used in chemodynamic therapy (CDT),^[^ ^8^ ^]^ as well as various combination therapies like photothermal therapy (PTT)^[^ ^9^ ^]^ and photodynamic therapy (PDT)^[^ ^10^ ^]^ due to its ability to generate toxic reactive oxygen species (ROS). Besides, it's been also found that calcium overload would increase tumor calcification and further mediate cell death.^[^ ^11^ ^]^ However, owing to its poor stability and limited targeting ability, the potential of CaO_2_ nanoparticles in radiotherapy sensitization toward in situ oral cancer has not been well investigated yet.

**Scheme 1:**
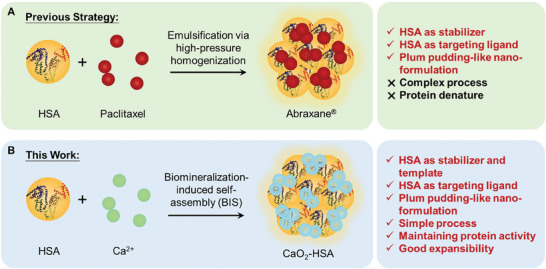
The similarities and differences between Abraxane and CaO_2_‐HSA in terms of preparation methods and properties.

Abraxane is a classic albumin‐bound paclitaxel nanomedicine, recognized as the first chemotherapy drug incorporating albumin approved by the U.S. Food and Drug Administration. It has been also the top‐selling nanomedicine in recent years. Abraxane is prepared using a high‐pressure homogenization method, resulting in nanoparticles ≈130 nm in size, with albumin and paclitaxel evenly distributed throughout. This structure not only enhances the stability of the formulation but also improves drug delivery to tumors through the endogenous natural albumin pathway.^[^ ^12^ ^]^ Inspired by the structure of a canonical nanodrug Abraxane, herein, as proof of concept, we describe a human serum albumin (HSA)‐modified CaO_2_ nanoparticles (CaO_2_‐HSA) system (**Scheme** **1**), and explore its application as the radiosensitizer toward in situ oral cancer. Specifically, HSA, with its binding capacity to calcium ions and being as an excellent stabilizer for nanoparticles, was used as a biological template for synthesizing the uniform and stable CaO_2_‐HSA co‐assembled nanoparticles via a biomineralization process. It also has a unique ability to improve pharmacokinetics and has become a promising candidate molecule for enhancing tumor tissue targeting.^[^ ^13^ ^]^ Subsequently, we validated that the anti‐tumor activity of CaO_2_‐HSA was derived from the calcium overload‐induced cell death and the radiosensitization effect, which was further verified by transcriptome sequencing. Furthermore, we constructed an animal model of in situ OSCC to evaluate the biosafety and efficacy of the CaO_2_‐HSA sensitization system by using a small animal radiation research platform and found that the CaO_2_‐HSA greatly improved the sensitizing enhancement ratio (SER), and exhibited a better final therapeutic efficacy in vivo.

## 2 Results

### 2.1 Synthesis and Characterization of CaO_2_‐HSA

**Figure 1:**
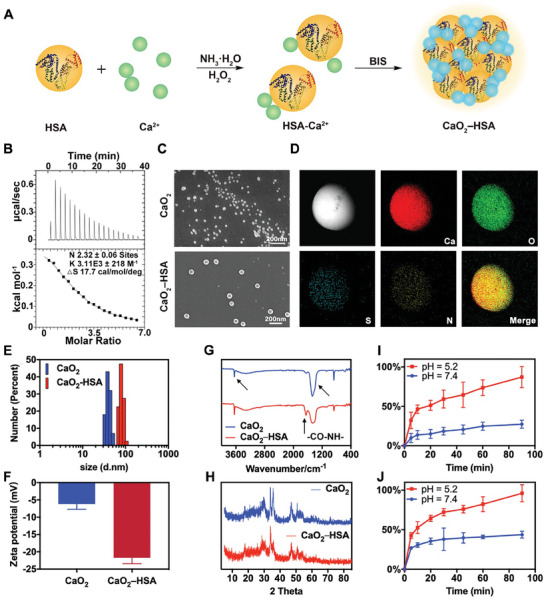
Preparation and characterization of CaO_2_‐HSA. A) Schematic diagram of CaO_2_‐HSA synthesis. B) Isothermal titration calorimetry was used to measure the binding force between calcium ions and HSA. The curve was obtained by titrating HSA with calcium chloride. C) Morphology assessment of CaO_2_ and CaO_2_‐HSA using scanning electron microscopy (SEM). D) EDS images showing the distribution for the elemental mapping of Calcium (red), Oxygen (green), Sulfur (cyan), and Nitrogen (yellow) in CaO_2_‐HSA. E,F) DLS measurements of hydrodynamic size (diameter) and zeta potential of CaO_2_ and CaO_2_‐HSA. Data presented as mean ± s.d. (n = 3). G) The Fourier transform infrared spectroscopy analysis. H) The XRD patterns of CaO_2_ and CaO_2_‐HSA, respectively. I) Calcium ion release curve of the CaO_2_‐HSA at pH 5.2 and 7.4 (n = 5). J) Hydrogen peroxide generation curve of the CaO_2_‐HSA at pH 5.2 and 7.4 (n = 5).

HSA‐modified calcium peroxide nanoparticles (CaO_2_‐HSA) can be easily and rapidly synthesized in aqueous phase and ice bath environment by biomineralization‐induced self‐assembly (BIS) (**Figure** **1**A). In short, calcium chloride was dissolved and then mixed with the biomineralization template HSA. HSA contains abundant active groups and can combine with calcium ions in the proportion of 1:2.32, which was verified by isothermal titration calorimetry (ITC) (Figure 1B). Subsequently, ammonia was introduced into the mixture to adjust the pH value, and hydrogen peroxide was slowly dropped in, triggering a reaction of

(1) $$\begin{array}{c} {\mathrm{CaCl}}_{2}\;+\;H_{2}O_{2}+2{\mathrm{NH}}_{3}\cdot H_{2}O+6H_{2}O\;\to {\mathrm{CaO}}_{2}\cdot 8H_{2}O+2{\mathrm{NH}}_{4}\mathrm{Cl}\;\;\; \end{array}$$

To confirm the desirable structure and function of the synthesized CaO_2_‐HSA, we then performed material characterization. The obtained products, as shown in scanning electron microscopy (SEM) (Figure 1C) and transmission electron microscopy (TEM) images (Figure S1, Supporting Information), are spherical nanoparticles with relatively uniform sizes. The CaO_2_‐HSA nanoparticles possessed a uniform lattice structure with a lattice fringe of d = 0.281 nm (Figure S2, Supporting Information). Through energy dispersive spectroscopy (EDS) and element mapping detection, it can be found that the characteristic elements N and S of HSA and Ca of CaO_2_ are homogeneously distributed over the nanoparticles (Figure 1D; Figure S3, Supporting Information), indicating the successful preparation based on biomineralization process. For further confirmation, the X‐ray photoelectron spectroscopy (XPS) of the nanoparticles was recorded (Figure S4, Supporting Information). The size of CaO_2_‐HSA is slightly larger than that of pure CaO_2_ nanoparticles, measured by DLS at 80.32 ± 11.55 nm and 55.85 ± 13.90 nm, respectively (Figure 1E; Figure S5, Supporting Information), and zeta potential decreased by ≈15 mV after the introduction of negative‐charged HSA (Figure 1F). The encapsulation efficiency of HSA is ≈36.5% (Figure S6, Supporting Information) measured by the Bradford method, and the protein content in CaO_2_‐HSA nanoparticles is ≈9.7% according to the thermogravimetric analysis (TGA) (Figure S7, Supporting Information). In the meanwhile, Fourier transform infrared (FTIR) absorption spectrum was performed, and the characteristic peaks 1471 and 3642 cm^−1^ corresponding to CaO_2_ and peaks between 1600 and 1700 cm^−1^ corresponding to amide bonds in HSA were observed (Figure 1G). The X‐ray diffraction (XRD) characterization also confirms that the diffraction peaks of the prepared product are consistent with those of the tetragonal CaO_2_ standard card (Figure 1H). Furthermore, the secondary structure of HSA in CaO_2_‐HSA remained constant, which was verified by circular dichroism (CD) (Figure S8, Supporting Information). Taken together, we determined that the nanoparticles are indeed composed of CaO_2_ nanocrystals cohered by native HSA. Next, the stability of CaO_2_‐HSA was evaluated in different pH buffer systems, with sodium acetate buffer (NaAc‐HAc, pH 5.2) simulating the slightly acidic tumor microenvironment and Tris•HCl buffer (pH 7.4) simulating a neutral environment. The release of calcium ions and hydrogen peroxide is much higher in acidic environments than in neutral environments (Figure 1I,J), indicating that nanoparticles can be triggered by pH at the effector site. At the same time, we also found that different buffer systems have significant differences in the degradation of nanoparticles. Storage in phosphate‐buffered saline (PBS) (pH 7.4) for 16 days resulted in less than 1% calcium leakage, indicating that the system would be more stable (Figure S9, Supporting Information).

### 2.2 Uptake of Nanoparticles Leads to Calcium Overload

**Figure 2:**
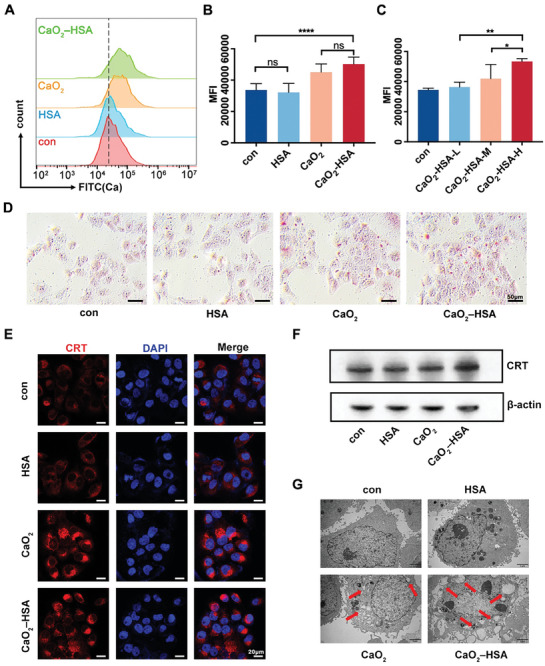
Uptake of nanoparticles leads to calcium overload. A,B) Intracellular calcium ion content after adding different groups of materials detected by flow cytometry. Data presented as mean ± s.d. (n = 6). C) Intracellular calcium ion content after adding different concentrations of CaO_2_‐HSA was detected by flow cytometry. Data presented as mean ± s.d. (n = 4). Statistical analysis is performed by one‐way ANOVA with Tukey's multiple comparisons test. (^*^ *p* < 0.05; ^**^ *p* < 0.01; ^****^ *p* < 0.0001; ns = no significance). D) Alizarin red staining showed the degree of calcium overload induced by different groups of materials. E) Immunofluorescence staining of the expression of CRT in CAL 27 cells stimulated by different materials. F) Western blot images of protein expression levels of CRT in CAL 27 cells after stimulation of various materials. G) The morphological changes of the subcellular organelles of CAL 27 cells after stimulation of various materials (observed through biological TEM) confirmed that CaO_2_‐HSA induced calcium overload leads to tumor death. Red arrows point out the vacuolization and apoptotic bodies.

To demonstrate whether nanoparticles can be internalized by cancer cells, we evaluated their cellular uptake using mouse oral squamous carcinoma cells CAL 27. Confocal laser scanning microscopy (CLSM) showed that fluorescence could be observed in cells 1 h after the addition of nanoparticles (Figure S10, Supporting Information), indicating that there was no obvious obstacle to the cellular uptake of nanoparticles. Then, the internalized nanoparticles can degrade in the cell, releasing large amounts of calcium ions. This was confirmed by flow cytometry using the Fluo‐4 probe (**Figure** **2**A). Compared with the control group and pure HSA group, CaO_2_ and CaO_2_‐HSA can significantly induce the up‐regulation of intracellular calcium ions (Figure 2B). It was also observed that with the increase of CaO_2_‐HSA addition, intracellular calcium ions would also increase in a concentration‐dependent manner (Figure 2C). As an effective second messenger, Ca^2+^ participates in a variety of physiological behaviors, such as neuronal excitation, protease expression, and bone morphogenesis.^[^ ^14^ ^]^ However, if the level of calcium ions in mitochondria increases, it will promote the process of mitochondrial electron transport chain (ETC) and interfere with the mitochondrial membrane potential, thus leading to Ca^2+^ overload and cell apoptosis.^[^ ^15^ ^]^ In cancer cells, mitochondrial Ca^2+^ homeostasis is highly fragile due to their dysfunctional calcium channels, and high levels of calcium (>500 nmol mg^−1^ mitochondria) will lead to the complete collapse of energy homeostasis, so they are more sensitive to Ca^2+^ regulation than normal cells.^[^ ^16^ ^]^ Therefore, introducing exogenous nanoparticles causing Ca^2+^ overload is an effective means of killing tumor cells. We used nanoparticles to conduct in vitro mineralization experiments on tumor cells and measured them by Alizarin staining, which is one of the gold standards for detecting calcified nodules. Compared with the control group and HSA group, more red calcified nodules were observed in the CaO_2_ and CaO_2_‐HSA groups (Figure 2D), further reflecting the occurrence of calcium overload.

It's been reported that calcium overload can also cause an immunogenic cell death (ICD) phenomenon.^[^ ^17^ ^]^ ICD is a special type of regulated cell death that occurs in dying tumor cells and activates adaptive immunity in immunocompetent hosts. One of the hallmarks of ICD is the increase in surface exposed calreticulin (CRT).^[^ ^18^ ^]^ Therefore, we detected the expression of CRT in tumor cells. By adding different groups of samples to the tumor cells, it can be clearly observed that the CRT of CaO_2_ and CaO_2_‐HSA groups have much higher fluorescence intensity than the HSA and control group under the confocal laser scanning microscopy (Figure 2E). By extracting total cell proteins for Western Blot experiments, we found that CaO_2_‐HSA group had the highest expression of CRT (Figure 2F).

Except for calreticulin exposure, immunogenic cell death in tumors is usually associated with HMGB1 and ATP release, and elevated HSP70/HSP90 expression. We tested these indicators in vitro to supplement the ICD data. Specifically, we stimulated CAL27 tumor cells with nanomaterials in vitro and analyzed the ATP content both within the cells and in the cellular supernatant. The results showed that the content of ATP in the tumor cells of CaO_2_ and CaO_2_‐HSA groups was decreased, while the content of ATP released into the supernatant was significantly increased (Figure S11, Supporting Information). We then used confocal fluorescence microscopy to observe the decrease of HMGB1 in the cells of CaO_2_ and CaO_2_‐HSA groups and the increase of HSP90 expression (Figure S12, Supporting Information). In addition, Western Blot results showed that the HSP90 expression of cells had the same trend, and the expression of HMGB1 protein was significantly down‐regulated in the CaO_2_‐HSA group (Figure S13, Supporting Information). At the same time, the expression of HSP70 on the cell surface was detected by flow cytometry, and the results showed that CaO_2_ and CaO_2_‐HSA groups had the highest fluorescence intensity (Figure S14, Supporting Information). Besides, to simulate the in vivo environment as much as possible, we used tumor cell culture supernatants to stimulate dendritic cells, one of the main effector cells of ICD. The results of flow cytometry showed that the supernatant of tumor cells stimulated by the CaO_2_ and CaO_2_‐HSA group could activate dendritic cells to the greatest extent, resulting in increased expression of CD80, CD86, and MHC II (Figure S15, Supporting Information). We believe that nanoparticles have a strong ability to induce ICD in tumor cells. At the microscopic level, after incubation with CaO_2_‐HSA, the tumor cell nucleus underwent pyknosis, resulting in vacuolization of the cell membrane and the formation of apoptotic bodies (Figure 2G). We emphasized the immunogenic death of tumor cells in the part of in vitro experiments and carried out cell‐level verification, including exposure of danger signals and activation of dendritic cells. All of these phenomena indicate that nanoparticles induce calcium overload in tumor cells, trigger ICD phenomenon, and ultimately lead to cell death.

### 2.3 The Generation of ROS and O_2_ Promotes the Effectiveness of Radiotherapy

**Figure 3:**
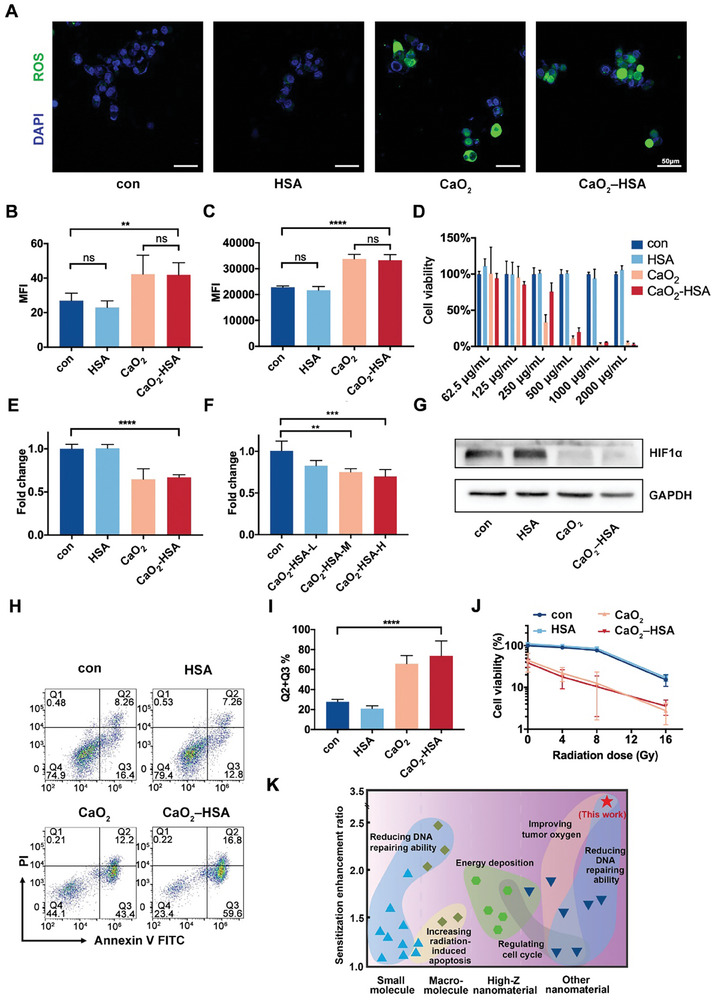
The generation of ROS and O_2_ promotes the effectiveness of radiotherapy. A) The amount of intracellular ROS after adding different materials detected by the DCFH‐DA fluorescent probe. Scale bars: 50 µm. B) Mean fluorescence intensity (MFI) of (A) was measured and analyzed (n = 6). C) Intracellular ROS production after adding different materials detected by flow cytometry (n = 6). D) Cytotoxicity assay of different groups using CCK8 method (n = 6). E) qRT‐PCR detection of HIF1α gene expression levels after stimulation with different materials under a hypoxic environment. F) qRT‐PCR detection of HIF1α gene expression levels after adding CaO_2_‐HSA with different concentrations under a hypoxic environment. G) Western blot detection of protein expression levels of HIF1α in CAL 27 cells after stimulation with various materials under a hypoxic environment. H,I) Apoptosis levels induced by different materials in hypoxic culture environments detected by flow cytometry (n = 6). J) Measurement of cell death caused by irradiation combined with material therapy in hypoxic environments using the CCK8 method (n = 6). Fitting cell survival rate using different doses of irradiation can calculate SER (defined as 15% cell survival rate). K) Comparison of SER values between the synthesized sensitizers in this study and common radiation sensitizers on the market/in research. CaO_2_‐HSA (red asterisk), small‐molecule radiosensitizers (azure triangles), macromolecular radiosensitizers (olive diamonds), high‐Z nanomaterial‐based radiosensitizers (green hexagons) and other nanomaterial‐based radiosensitizers (indigo triangles). They are also classified into different groups according to the mechanism of radiosensitization. Details of the aforementioned sensitizers are listed in Table S2 (Supporting Information). Data presented as mean ± s.d. Statistical analysis is performed by one‐way ANOVA with Tukey's multiple comparisons test. (^**^ *p* < 0.01; ^***^ *p* < 0.001; ^****^ *p* < 0.0001; ns = no significance).

With the dissolution of nanoparticles inside cells, in addition to releasing calcium ions, they also released hydrogen peroxide and increased the content of ROS in cells, which was monitored through CLSM and DCFH‐DA probes. The control group and HSA group showed weak signals, while the CaO_2_ and CaO_2_‐HSA group showed strong intracellular green fluorescence, indicating an increase in intracellular ROS (**Figure** **3**A,B). The consistent results were obtained using flow cytometry (Figure 3C). It is worth noting that there may be a self‐amplifying circuit between the increased intracellular calcium ions and ROS. On the one hand, the stimulation of Ca^2+^ on the tricarboxylic acid cycle (TCA cycle) causes the entire mitochondria to work faster and consume more O_2_ to increase ROS output. Meanwhile, Ca^2+^ can interfere with the antioxidant status of mitochondria, and mitochondria exposed to Ca^2+^ may produce more ROS due to a decrease in GSH levels.^[^ ^19^ ^]^ On the other hand, ROS increase can stimulate Ca^2+^ influx by regulating plasma membrane Ca^2+^ channels (such as VDCC, TRP, and SOCE), intracellular calcium channels (such as RyR and IP3R), and Ca^2+^ ATPase.^[^ ^20^ ^]^ In summary, both calcium overload and increased ROS are beneficial for tumor killing. CCK8 method was used to detect the cell activity after incubation of different groups of nanoparticles. The results showed that the IC50 value of CaO_2_‐HSA was 327 µg mL^−1^ (Figure 3D), which was slightly higher than that of the pure CaO_2_ group. This may be due to the addition of HSA, which to some extent increases biosafety, but the difference is only limited to a concentration of 250 µg mL^−1^.

To mimic the hypoxic microenvironment inside the tumor, we applied nanoparticles to cells under a hypoxia incubator (1% O_2_). Given that both CaO_2_ itself and H_2_O_2_ produced by CaO_2_ can generate oxygen in the catalase‐independent or catalase‐dependent way in tumors and increase dissolved oxygen content^[^ ^21^ ^]^ (Figure S16, Supporting Information), thus the hypoxia condition would be reversed. qRT‐PCR and Western Blot were exploited to verify the changes in the key indicator HIF1α of hypoxia (Figure 3E–G). From the results, it can be seen that the gene and protein expression of HIF1α decreased in the CaO_2_ and CaO_2_‐HSA group while exhibiting a dose‐dependent manner. Therefore, it can be demonstrated that the hypoxia phenomenon of cells is inhibited after the application of nanoparticles. Furthermore, we investigated whether the reversed hypoxic microenvironment could significantly improve radiation sensitivity. Encouragingly, in the tumor cells treated with 7.5 Gy radiation under hypoxia condition, the CaO_2_‐HSA exhibited 2.65 times more potent than the control group. (Figure 3H,I). The CCK8 method was used to measure cell activity after material stimulation and radiation irradiation, and the sensitivity enhancement ratio (SER) of CaO_2_‐HSA was calculated to be 3.47 (Figure 3J). The effect is superior to common radiosensitizers on the market and other studies on radiation sensitization (Figure 3K).^[^ ^22^ ^]^

### 2.4 Transcriptome RNA Sequencing Reveals Mechanisms of Radiotherapy Sensitization

**Figure 4:**
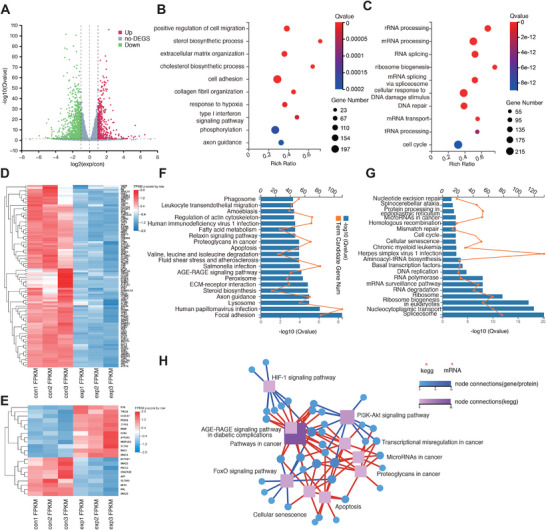
Transcriptome RNA sequencing reveals mechanisms of radiotherapy sensitization. A) Volcano plot of differentially expressed genes of CAL 27 cells activated by CaO_2_‐HSA under a hypoxic environment. B,C) Gene ontology‐biological process analysis of DEGs of control group (B) and CaO_2_‐HSA group (C). D) Heat map shows the expression levels of 94 genes related to hypoxia in different groups. E) Heat map shows the expression levels of 21 genes related to calcium ion homeostasis and transportation in different groups. F) KEGG pathway analysis of upregulated genes of CAL 27 cells in the control group. G) KEGG pathway analysis of upregulated genes in CaO_2_‐HSA group. H) KEGG pathway relationship network obtained through hypoxia related gene analysis.

In order to further clarify the mechanisms of improved radiation sensitivity of CaO_2_‐HSA on tumor cells, we conducted transcriptome RNA sequencing analysis between the control group (PBS) and the experimental group (CaO_2_‐HSA). Two groups of CAL 27 cells were treated with 7.5 Gy irradiation under hypoxia conditions simultaneously, with three duplicate samples in each group. There were 7254 genes with significant expression differences between the CaO_2_‐HSA and control groups, involving 3700 upregulated genes and 3554 down‐regulated genes (**Figure** **4**A). The heatmap shows differential expression genes (DEGs) in the experimental and control groups (Figure S17, Supporting Information). Based on those differences, Gene Ontology (GO) analysis and Kyoto Encyclopedia of Genes and Genomes (KEGG) analysis were carried out to clarify the biological functions of altered mRNA and the corresponding pathways (Figures S18 and S19, Supporting Information). GO analysis shows that the hypoxia response in the control group has significant changes, which is in line with our expectations (Figure 4B). While in the experimental group, GO analysis shows that the biological processes of DNA damage and repair, as well as changes in RNA related biological processes, are more prominent (Figure 4C). Because the radiation would irreversibly damage the DNA of tumor cells, leading to cell apoptosis and death. Next, we conducted cluster analysis on all the 94 genes related to hypoxia in the database. The results show that the expression level of the control group is higher than that of the experimental group (Figure 4D), indicating that CaO_2_‐HSA nanoparticles significantly reversed cell hypoxia levels. On the other hand, cluster analysis was conducted on a total of 223 genes related to DNA damage and repair in the database. The heatmap shows that the expression levels of these genes in the experimental group are significantly higher than those in the control group, indicating that tumor cells are severely damaged after radiotherapy (Figure S20, Supporting Information). In addition, gene analysis of calcium ion transport and regulation shows that the expression level in the experimental group is also higher than that in the control group (Figure 4E).

For further mechanism analysis, the first 20 pathways based on KEGG analysis were constructed. Compared with the control group, the differentially expressed genes in the CaO_2_‐HSA group are rich in DNA damage and repair related pathways, including cell cycle, mismatch repair, and nucleotide exclusion repair, similar to GO analysis (Figure 4F,G). KEGG pathway relationship network analysis was then conducted on 94 DEGs related to hypoxia, and it's found that they are closely related to HIF‐1 pathway, PI3K‐AKT pathway, FoxO pathway, and microRNA (Figure 4H), which can further serve as research targets for handling tumor hypoxia. Based on the above evidence, it has once again been verified that the CaO_2_‐HSA nanoparticles can reshape the hypoxic microenvironment of tumor cells and enhance the sensitivity and efficacy of radiotherapy.

### 2.5 In Vivo Antitumor Efficacy of CaO_2_‐HSA‐Based Radiosensitizer for In Situ Oral Cancer

**Figure 5:**
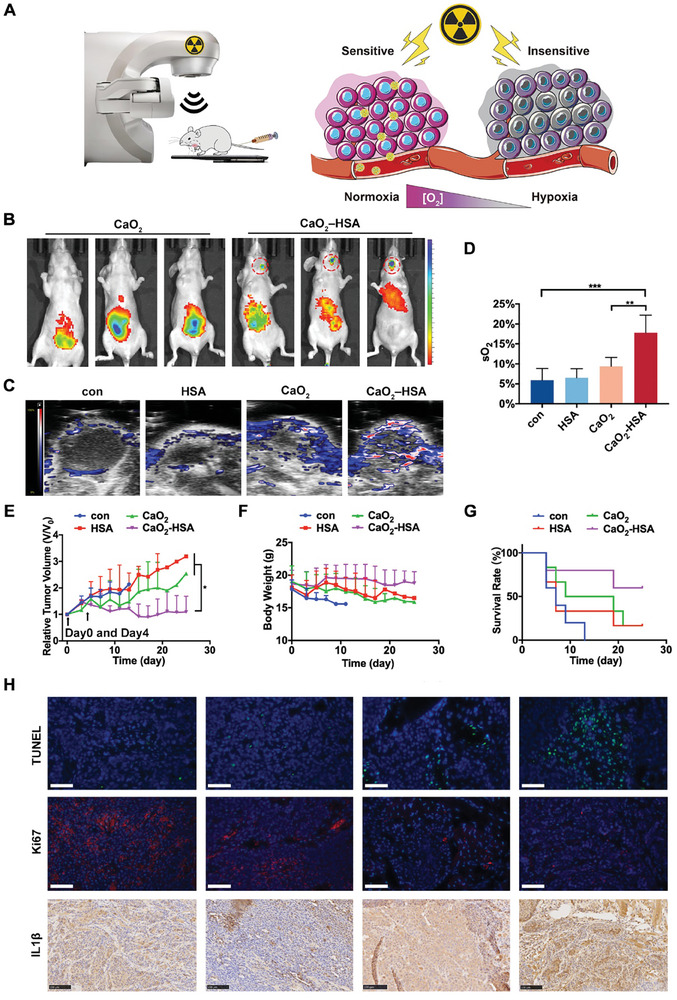
In vivo antitumor efficacy of CaO_2_‐HSA‐based radiosensitizer for in situ oral cancer. A) Schematic diagram of CaO_2_‐HSA as a radiosensitizer for treatment. B) Fluorescence images of mice injected with CaO_2_ and CaO_2_‐HSA. CaO_2_‐HSA migration at a region of interest (ROI, highlighted with red circles) was shown. C) Photoacoustic (PA) imaging of CAL 27 tumor and peritumor blood oxygen saturation (sO_2_) levels. D) Quantification of peritumor sO_2_ levels. Data presented as mean ± s.d. (n = 5–6). E) The profiles of tumor volume variation. Data presented as mean ± s.d. (n = 5–6). Black arrows mean the days of administration. F) Mice body weights after various treatments (n = 5–6). G) Survival curves of mice receiving different treatments (n = 5–6). H) Local section staining images of tumor tissue. TUNEL (green) staining and Ki67 (red) staining of tumor sections were observed by fluorescence microscopy. Cell nuclei were stained with DAPI (blue). The expression of IL‐1β was evaluated by immunohistochemical staining. Scale bars: 100 µm. Statistical analysis is performed by one‐way ANOVA with Tukey's multiple comparisons test. (^*^ *p* < 0.05; ^**^ *p* < 0.01; ^***^ *p* < 0.001).

Encouraged by in vitro results, we further explored in vivo antitumor efficacy of CaO_2_‐HSA‐based radiosensitizer for in situ oral cancer (**Figure** **5**A). First, we assessed the tumor targeting ability of CaO_2_‐HSA in OSCC‐bearing mice. The OSCC model was established by injecting CAL 27 cells into the sublingual area of immunodeficient mice via their mandible hyoid muscle in an extraoral direction. 100 µL Cytate fluorescent labeled CaO_2_ and CaO_2_‐HSA nanoparticles were injected through the tail vein. IVIS small animal imaging system was performed on mice after 4 h. The results showed that CaO_2_‐HSA fluorescent nanoparticles can accumulate at the tumor site at the base of the tongue, with significantly higher fluorescent intensity than that of the CaO_2_ group (Figure 5B), confirming the excellent properties of CaO_2_‐HSA for in vivo tumor targeting delivery. It's been reported that HSA‐based nanoparticles can target tumors through the enhanced permeability and retention (EPR) effect, with receptor‐mediated mechanisms further enhancing their tumor specificity.^[^ ^13^ ^]^ After 12 h, imaging was performed on the main organs of mice to unveil the biodistribution of nanoparticles (Figure S21, Supporting Information). To further evaluate the potential damage to tissues caused by nanoparticles, we took samples from mice injected with nanoparticles 14 days later and stained each major organ with hematoxylin and eosin (H&E). No significant damage was observed in the heart, liver, spleen, lung or kidney of mice (Figure S22, Supporting Information). Meanwhile, both clinical biochemistry and hematological parameters remained within the safe range (Figures S23 and S24, Supporting Information). The hemolysis test also indicates that the concentration of CaO_2_‐HSA used in both in vitro and in vivo experiments does not cause hemolysis, unless at an extremely high concentration (Figure S25, Supporting Information). At the same time, it can be found that even at high concentrations, the biocompatibility of CaO_2_‐HSA is better than that of the CaO_2_ itself.

To further elucidate the efficacy of nanoparticles on tumors under radiation, PBS, HSA, CaO_2_, and CaO_2_‐HSA nanoparticles were injected into OSCC‐bearing mice through the tail vein at a dose of 25 mg kg^−1^. After 4 h, the mice were irradiated with 8 Gy with a dedicated small animal radiotherapy device. Photoacoustic tomography was exploited to monitor the tumor oxygen saturation (sO_2_) during the treatment, and the results showed that the oxygen content inside the tumor in the CaO_2_‐HSA group was 1.89 times that of the CaO_2_ group alone and 3.01 times that of the control group (Figure 5C,D). The generation of oxygen has been proven to play an important role in the effectiveness of radiotherapy.^[^ ^23^ ^]^ The body weight, tumor size, and survival status of mice were monitored every other day after the initial treatment (Figure 5E–G). Given that the increase in tumor volume may compress the trachea and esophagus, all remaining mice were sacrificed on the 25th day of treatment. The tumor tissue located at the base of the tongue was taken for photography, which can intuitively demonstrate the effective anti‐tumor effect of CaO_2_‐HSA nanoparticles (Figure S26, Supporting Information). In terms of weight, due to the fact that the tumor is located at the base of the tongue, it may have a slight impact on the mice's diet, resulting in a small amount of weight loss in each group. However, the CaO_2_‐HSA group ultimately maintained the highest body weight, indicating an improvement in the quality of life of mice. Therefore, CaO_2_‐HSA nanoparticles showed a significantly enhanced tumor inhibition rate and the highest survival rate in mice.

The superior antitumor efficacy of CaO_2_‐HSA was further verified by histological analysis (Figure 5H). H&E staining was used to determine the infiltrating area of the tumor (Figure S27, Supporting Information). TUNEL staining at the tumor site indicated that the CaO_2_‐HSA group had the highest level of cell apoptosis (Figure S28, Supporting Information). Ki67 staining showed that the control group had the strongest tumor proliferation ability and strong growth and invasion potential, while the CaO_2_‐HSA group was only at the lowest level (Figure S29, Supporting Information). We also detected the expression of ICD indicators including CRT and HSP90 in tumor tissue samples, and immunohistochemical staining results also showed that CaO_2_ and CaO_2_‐HSA groups had the strongest expression (Figure S30, Supporting Information). Meanwhile, we used immunohistochemical staining to analyze the expression of pro‐inflammatory factors in the local tumor area. The levels of IL‐1β and IL‐18 (Figure S31, Supporting Information) are both the highest in the CaO_2_‐HSA group, indicating that the application of nanoparticles enhanced local inflammatory infiltration and was beneficial for the subsequent anti‐tumor immunity mediated by innate immune cells (such as tumor‐associated macrophages and NK cells). In summary, CaO_2_‐HSA‐based radiosensitizers exhibit excellent in vivo anti‐tumor efficacy.

## 3 Discussion

An ideal radiosensitizer for hypoxia reversal should possess the following properties: 1) adequate oxygen production performance; 2) good biocompatibility; 3) tumor targeting ability; 4) stability and satisfactory bioavailability; 5) relatively simple composition and facile preparation process for a better bench‐to‐bedside translation. Based on the results, we hold the view that CaO_2_‐HSA can meet the above five requirements of radiosensitizer well. As far as we know, CaO_2_ nanoparticles have not been applied to radiosensitization of orthotopic implantation OSCC model in previous literature studies, although very few works reported the CaO_2_‐containing untargeted system for radiosensitization in subcutaneous tumor models.^[^ ^24^ ^]^ Inspired by the elegant structure of classic Abraxane where HSA acts as both the targeting ligand for SPARC and the stabilizer for hydrophobic paclitaxel,^[^ ^25^ ^]^ we constructed the CaO_2_‐HSA nano‐formulation via a biomineralization process instead of high‐pressure homogenization adopted by Abraxane, which leads to protein denature. On the one hand, HSA can stabilize the CaO_2_ nanocrystals during the nucleation and growth; on the other hand, HSA would also be induced to assemble along with the crystallization of CaO_2_, gradually forming a uniform organic‐inorganic hybrid structure, in which the conformation and biological activity of HSA can be well maintained.

It should be pointed out that CaO_2_‐HSA exhibited a high inhibition rate against tumor growth though, it cannot completely abolish the tumor. It is reasonable in view of the fact that radiotherapy would not be utilized as an isolated treatment toward cancer in clinics commonly, but be combined with surgery, chemotherapy, and other therapeutics as an auxiliary treatment.^[^ ^26^ ^]^

## 4 Conclusion

In conclusion, as proof‐of‐concept research, we have successfully tailored the CaO_2_‐HSA system as the radiosensitizer for in situ oral cancer radiotherapy. In the Abraxane‐like structure of CaO_2_‐HSA, CaO_2_ nanocrystals were cohered by native HSA resulting from the biomineralization process, allowing the hybrid nano‐formulation to achieve great stability, biocompatibility, and tumor targeting ability. It is worth noting that the SER value of CaO_2_‐HSA is much higher than that of sodium glycididazole (reportedly less than 1.5 ^[^ ^22b,f^ ^]^), the only approval radiosensitizer in China, and it's ascribed to the synergistic effect of calcium ion interference to cause ICD phenomenon and oxygen production to reverse the hypoxia that was confirmed by transcriptome RNA sequencing. Potential mechanisms underlying the cytotoxic effects of CaO_2_‐HSA on tumor cells encompass calcium overload, release of ROS, and the induction of ICD. With in situ OSCC mice model, the excellent anti‐tumor efficacy and biosafety of CaO_2_‐HSA have been validated. Last but not the least, compared starkly with other heavy metal‐based radiosensitizers, the biocompatibility and facile preparation property of CaO_2_‐HSA may thus create a new opportunity for translational medicine. In the future work, combining CaO_2_‐HSA with various new therapies will be comprehensively considered to further enhance the efficacy of oral cancer therapy.

## Conflict of Interest

The authors declare no conflict of interest.

## Supporting information

Supporting Information

## Data Availability

The data that support the findings of this study are available in the supplementary material of this article.
