# Supplementary material for: “Abraxane‐Like” Radiosensitizer for In Situ Oral Cancer Therapy
Source: Adv Sci (Weinh). 2024 Jul 7;11(34):2309569. doi: 10.1002/advs.202309569 (PMC11425904; doi:10.1002/advs.202309569)
Supplement: Supplementary file 1 — Supporting Information [file ADVS-11-2309569-s001.docx]

Supporting Information

“Abraxane-like” Radiosensitizer for *in situ* Oral Cancer Therapy

Zijian Gong, Yixuan Fu, Yuan Gao, Fei Jiao, Qinzhi Su, Xiao Sang, Binglin Chen, Xuliang Deng* and Xinyu Liu*

**Outline**

[Methods 2](#_Toc166482270)

[Chemicals and reagents 2](#_Toc166482271)

[Characterization 2](#_Toc166482272)

[Synthesis of CaO_2_-HSA 2](#_Toc166482273)

[pH sensitivity evaluation 2](#_Toc166482274)

[Immunofluorescent staining 3](#_Toc166482275)

[Flow Cytometry 3](#_Toc166482276)

[ATP(adenosine 5'-triphosphate) content detection 4](#_Toc166482277)

[Alizarin red staining 4](#_Toc166482278)

[Western Blot 4](#_Toc166482279)

[Cytotoxicity test induced by nanomaterials 5](#_Toc166482280)

[RNA transcriptome sequencing 5](#_Toc166482281)

[Real-time Quantitative polymerase chain reaction 5](#_Toc166482282)

[Apoptosis induced by nanoparticles in radiotherapy combined with hypoxia 6](#_Toc166482283)

[Cytotoxicity test induced by nanomaterials and irradiation 6](#_Toc166482284)

[*In vivo* treatment of oral cancer mice 6](#_Toc166482285)

[Tissue staining 7](#_Toc166482286)

[Oxy-Hemo photoacoustic (PA) imaging 7](#_Toc166482287)

[Live imaging of small animals 7](#_Toc166482288)

[Hemolysis test 7](#_Toc166482289)

[Biosafety Testing 8](#_Toc166482290)

[Statistical analysis 8](#_Toc166482291)

[Data available 8](#_Toc166482292)

[Supplementary figures 9](#_Toc166482293)

# Methods

## Chemicals and reagents

Human serum albumin was purchased from Wuhan Healthgen Biotechnology Corp. Polyvinylpyrrolidone-K30 was bought from Beijing OKA Biotechnology Co., Ltd. Cypate was purchased from Shanghai Bidepharmatech Co., Ltd. Calcium chloride, ammonia, hydrogen peroxide and all other reagents were purchased from Sinopharm Chemical Reagent Co. Ltd and of analytical grade.

## Characterization

The morphologies of nanomaterials were recorded with a transmission electron microscope (TEM) with a working voltage of 100 kV (JEM-1400, JEOL). The SEM images were obtained on a field-emission scanning electron microscope (Zeiss SIGMA). The average particle size and Zeta potential were measured with Zetasizer Nano ZS90 (Malvern). X-ray diffraction (XRD) patterns were collected on D8 advance (Bruker) at room temperature. Thermal Analysis was conducted by STA 449C (NETZSCH). TECNAI G2 F30 transmission electron microscope was used for element mapping test and High-resolution TEM image. XPS spectra was conducted by EscaLab 250Xi (Thermo). ITC experiments were performed with MicroCal iTC200 (Malvern). The testing of circular dichroism was carried out using the Jasco J-1500 instrument. The equipment model used for animal radiation was X-RAD 320 Biological Irradiator (Precision X-ray, Inc., North Branford, CT) and the experimental parameter was 160 kV/12.5 mA to irradiate different doses of X-rays.

## Synthesis of CaO_2_-HSA

Weigh 0.1 g of calcium chloride, 0.35 g of PVP and 50 mg of HSA into a round-bottom flask, add 15 mL of cold double distilled water, and dissolve them with ultrasound to make them disperse evenly. Afterwards, stir under ice bath conditions and add 1 mL of ammonia water to it. Slowly add 30% hydrogen peroxide to the system at a rate of 50 μL/min for four minutes. Continue stirring for five minutes, collect the liquid into a centrifuge tube, and centrifuge at 8000 rpm for five minutes. Wash three times with double distilled water to obtain CaO_2_-HSA nanoparticles. For the synthesis of calcium peroxide, HSA is not added to the raw material, and the other steps are the same as CaO_2_-HSA synthesis.

## pH sensitivity evaluation

Place 15 mg of synthesized nanoparticles in an EP tube, add 2 mL of buffer solution (0.2 M NaAc (pH = 5.2) or 0.2 M Tris•HCl (pH = 7.4)) and mix well. Centrifuge at 9000 rpm for 1 minute after a certain time interval, and collect 50 microliters of supernatant for subsequent testing. For the detection of calcium ion release and hydrogen peroxide release, follow the instructions separately (S1063S, Beyotime; BC3595, Solarbio) and draw the release curve. For long-term stability testing, nanoparticles were placed in PBS, centrifuged every one or two days and collected a portion of the supernatant. Calcium ion release is detected using a calcium ion content colorimetric assay kit (S1063, Beyotime).

## Immunofluorescent staining

The CAL 27 cell line was obtained from the American Type Culture Collection (ATCC) and cultured in Dulbecco’s modified Eagle’s medium (Procell Life Science&Technology Co.,Ltd.) with 10% fetal bovine serum (Procell Life Science & Technology Co.,Ltd.) at 37 °C in a humidified 5% CO_2_ atmosphere. In 10 mM sodium bicarbonate solution, Cy5 was added to label nanoparticles, while several drops of anti-fluorescence quencher (P0128S, Beyotime) were added, and the precipitate was collected by washing and centrifugation to obtain Cy5 labeled CaO_2_-HSA.

*Detection of nanoparticle internalization:* CAL 27 cells were seeded in confocal dishes and used when the cell density was at 80%. Samples were collected after one hour of incubation with fluorescently labeled nanoparticles. Cells were fixed by adding 4% paraformaldehyde for 15 min at room temperature and washed three times with PBS. After that, the cells were drilled with 0.5% Triton-X-100 at room temperature for 15 min and washed three times with PBS. FITC-phalloidin (1:200, CA1620, Solarbio) was diluted with 1% BSA, incubated at 37 ° C for 1 h, and washed three times with PBS. DAPI (C1002, Beyotime) was added to stain the nuclei for 10 min. Finally, the images were obtained by fluorescence confocal microscope (Zeiss LSM880).

*Detection of CRT/HSP90/HMGB1 expression:* CAL 27 cells were seeded in confocal dishes and used when the cell density was at 70%. Samples were collected after 24 hours of incubation with nanoparticles. Cells were fixed by adding 4% paraformaldehyde for 15 min at room temperature and washed three times with PBS. After that, the cells were drilled with 0.5% Triton-X-100 at room temperature for 15 min and washed three times with PBS. Cells were blocked using 1% BSA solution at 37 °C for one hour, after which CRT antibody (1:200, 27298-1-AP, Proteintech) / HSP90β antibody (1:400, A23489, Abclonal)/ HMGB1 antibody (1:200, A19529, Abclonal) was added and incubated overnight at 4 °C. The next day, they were washed three times with PBS and incubated with 594-conjugated Goat anti-rabbit IgG (1:200, SA00013-4, Proteintech) or 488-conjugated Goat anti-rabbit IgG (1:200, SA00013-2, Proteintech) at 37 °C for one hour. After washing three times with PBS, DAPI (C1002, Beyotime) was used to stain the nuclei for 10 min.

*Detection of intracellular ROS content.* CAL 27 cells were seeded in confocal dishes and used when the cell density was at 80%. Follow the instructions of ROS Assay Kit (S0033S, Beyotime). Specifically, ROS probe (1:1000) was first loaded and incubated for 20 min. The nanoparticles were added for 30 min after PBS washing for three times. After that, the cells were washed three times with PBS, and the nuclei were stained with Hoechst 33342 (C1027, Beyotime) for 10 min.

## Flow Cytometry

*Detection of intracellular calcium content.* CAL 27 cells were seeded in six-well plates and used when the cell density was at 80%. Samples were collected after one hour of incubation with nanoparticles. Prepare fluo-4 working solution (1:1000, S1060, Beyotime), add 50 μL to each sample and mix well, and incubate at 37 ℃ for 30 min. After that, the cells were washed by centrifugation, collected into flow tubes, and the fluorescence intensity of FITC channel was detected by flow cytometry (Cytoflex).

*Detection of intracellular ROS content.* CAL 27 cells were seeded in six-well plates and used when the cell density was at 80%. Follow the instructions of ROS Assay Kit (S0033S, Beyotime). Specifically, ROS probe (1:1000) was first loaded and incubated for 20 min. The nanoparticles were added for 30 min after PBS washing for three times. After that, the cells were collected into flow tubes.

*Detection of HSP70 expression in cells.* CAL 27 cells were seeded in six-well plates and used when the cell density was at 60%. Samples were collected after 24 hours of incubation with nanoparticles. Prepare Alexa Fluor® 488 anti-Hsp70 working solution (1:200, 648003, BioLegend), add 50 μL to each sample and mix well, and incubate at 4 ℃ for 30 min. After that, the cells were washed by centrifugation, collected into flow tubes, and the fluorescence intensity was detected by flow cytometry (Cytoflex).

*Detection of Dendritic cell activation markers.* SCC7 cells were seeded in six-well plates and used when the cell density was at 60%. After different materials were co-cultured with cells for 24 hours, the supernatant was collected. DC2.4 cells were seeded in six-well plates and used when the cell density was at 60%. The supernatant of different groups was added and co-cultured for 24 hours, and then cells were collected. Prepare antibody working solution (1:200, PE anti-mouse CD86, 159203, BioLegend; 1:200, FITC anti-mouse I-A/I-E, 107605, BioLegend; 1:200, APC anti-mouse CD80, 104713, BioLegend), add 50 μL to each sample and mix well, and incubate at 4 ℃ for 30 min. After that, the cells were washed by centrifugation, collected into flow tubes, and the fluorescence intensity was detected by flow cytometry (Cytoflex).

## ATP (adenosine 5'-triphosphate) content detection

CAL 27 cells were seeded in 6-well plates and used when the cell density was at 70%. The cells were stimulated by nanoparticles for 24 h. The cell supernatant is centrifuged and collected. The cells are treated with lysate and collected. All operations are performed according to kit instructions (Enhanced ATP Assay Kit, S0027, Beyotime). RLU values were determined using a black 96-well plate in a Multifunctional Microplate Reader (SpectraMaxiD5, Molecular Devices).

## Alizarin red staining

CAL 27 cells were seeded in 12-well plates and used when the cell density was at 70%. The cells were stimulated by nanoparticles for 24 h. Fixed with 4% paraformaldehyde for 15 min at room temperature and washed three times with PBS. Add 500 μL alizarin red dye solution (Procell Life Science & Technology Co., Ltd.), incubate at room temperature for 30min, and wash three times with PBS. Images were taken under a microscope.

## Western Blot

CAL 27 cells were seeded in 6-well plates and used when the cell density was at 60%. The hypoxic incubator conditions were set as 1% oxygen and 5% carbon dioxide, and the cells were incubated in it overnight. The cells were then stimulated by nanoparticles for 24 h. Cells were lysed using RIPA lysate (P0013B, Beyotime) containing PMSF (ST507, Beyotime) and collected into EP tubes. Next, ultrasonic treatment was carried out until the liquid fluidity was strong. Centrifuge at 12000 rpm for 10 min at 4 °C to remove the precipitate. The protein concentration of the supernatant was determined using BCA detection kit. Add quantitative 6 × protein loading buffer (P0285, Beyotime) to the sample, mix well, and treat with 95 ℃ in a metal bath for 10 minutes. Use 10% or 12% polyacrylamide gel and apply 10 μL sample to each well. Electrophoresis was performed at constant voltage 160 V for 45 min. Next, the membrane was rotated to form a sandwich structure. Electrophoresis was performed at a constant flow of 200 mA for 120 min under ice bath conditions. After that, 5% nonfat milk was used for blocking at room temperature for 1 hour. Incubate overnight at 4 °C using antibody (1:10000 for anti-GAPDH (AC002, Abclonal), 1:75000 for Anti-β-actin (AC026, Abclonal), 1:1000 for Anti-HIF1α (20960-1-AP, Proteintech), 1:1000 for Anti-CRT (27298-1-AP, Proteintech), 1:2000 for HSP90β antibody (A23489, Abclonal), 1:1000 for HMGB1 antibody (A19529, Abclonal)). The next day, the membrane was washed three times with TBST and incubated with HRP-conjugated secondary antibody (BL001A, BL003A, Biosharp) for 1 hour at room temperature. Wash with TBST for four times, and prepare the ECL Western Blotting Substrate (PE0010, Solarbio) for exposure.

## Cytotoxicity test induced by nanomaterials

CAL 27 cells were seeded in 96 well plates and incubated with nanomaterials for 24 h. Cytotoxicity was measured according to the instructions of CCK8 Assay kit (BS350A, Biosharp). In brief, remove the old cell culture medium, prepare the fresh medium and CCK8 solution in the ratio of 10:1, and add 200 μL in each well. Incubate in the incubator for 1 hour in the dark, and use a microplate reader to read the absorbance value at 450 nm. Untreated cells were used as control values to calculate cytotoxicity.

## RNA transcriptome sequencing

CAL 27 cells were seeded in 6-well plates and used when the cell density was at 60%. The hypoxic incubator conditions were set as 1% oxygen and 5% carbon dioxide, and the cells were incubated in it overnight. Cells in the experimental group were co-cultured with nanomaterials for 24 h, while the control group was added with the same amount of PBS. Operate on ice during the whole process of sample extraction. First, the cells were washed three times with PBS, 1 mL of Trizol was added to the dish and left to stand for 3 min. Then collect the solution to the cryopreservation tube and snap freeze it with liquid nitrogen. The samples were sent to BGI using dry ice for subsequent RNA extraction, library building, computerization and analysis. Differential gene expression analysis was conducted in DESeq2 and significant differentiation genes were identified when adjusted P < 0.05 and log2FC >= 1. And then significant differential genes were performed for KEGG gene enrichment and gene ontology (GO) enrichment. The visualization of the results was made using Dr. Tom system (BGI-Shenzhen, China).

## Real-time Quantitative polymerase chain reaction

CAL 27 cells were seeded in 6-well plates and used when the cell density was at 60%. The hypoxic incubator conditions were set as 1% oxygen and 5% carbon dioxide, and the cells were incubated in it overnight. Cells in the experimental group were co-cultured with nanomaterials for 24 h, while the control group was added with the same amount of PBS. RNA extraction was performed completely according to the kit instructions (FastPure Cell/Tissue Total RNA Isolation Kit V2, RC112, Vazyme). Nanodrop was used to evaluate RNA concentration and quality. The acquisition of cDNA and the construction of qRT-PCR system were carried out using kits (Q712, Vazyme; R323, Vazyme) respectively. QuantStudio™ 3 System (Thermo Fisher) was used for testing. The primer sequences are shown in Table S1

Table S1

| Gene | Primer sequences (F, forward; R, reverse) |
| --- | --- |
| HIF1α | F: 5’- GAACGTCGAAAAGAAAAGTCTCG-3’ |
|  | R: 5’- CCTTATCAAGATGCGAACTCACA-3’ |
| GAPDH | F: 5’- GCACCGTCAAGGCTGAGAAC-3’ |
|  | R: 5’- TGGTGAAGACGCCAGTGGA-3’ |

## Apoptosis induced by nanoparticles in radiotherapy combined with hypoxia

CAL 27 cells were seeded in 6-well plates and used when the cell density was at 60%. The hypoxic incubator conditions were set as 1% oxygen and 5% carbon dioxide, and the cells were incubated in it overnight. The experimental group was stimulated with the nanomaterials for two hours, while the control group was stimulated with the same amount of PBS. After that, the six-well plate was put into the radiotherapy instrument for 7.5 Gy irradiation. Transfer to incubator for further incubation for 24 h. Subsequently, the level of apoptosis was detected by flow cytometry. When collecting samples, the supernatant was retained, the cells were digested with trypsin without EDTA, and the cells were collected by centrifugation. After washing twice with PBS, the binding solution, Annexin V-FITC and PI were added for staining (CA1020, Solarbio). Rapid detection using flow cytometry (Cytoflex).

## Cytotoxicity test induced by nanomaterials and irradiation

CAL 27 cells were seeded in 96 well plates for 24 h. The first 12 hours were cultured in a normal oxygen content incubator, and the next 12 hours were cultured in a hypoxic incubator (1% O_2_). After culturing with different nanoparticles for two hours, different doses of irradiation were applied. Concentrations of different nanoparticles used in this experiment were set at the IC_50_ value based on the CCK8 assay results. Then continue hypoxia cultivation for 24 hours. Cytotoxicity was measured according to the instructions of CCK8 Assay kit (BS350A, Biosharp).

## *In vivo* treatment of oral cancer mice

30 six-week-old female Balb/c nude mice were obtained from Beijing Vital River Laboratory Animal Technology Co., Ltd. All animal experiments were approved by the Ethics Committee of Beijing University (ethical approval number LA2022274). The animal experiment process complied with all relevant ethics. Mice were anesthetized with isoflurane, and 50 microliters of cells were injected into the root of tongue through submaxillary injection, with a total amount of about 6 × 10^6^ cells. After about 3 weeks, the treatment was started when the tumor volume reached 100 mm^3^. Different groups of nanoparticles were injected through the tail vein. Afterwards, a dedicated small animal radiation irradiator was used for irradiation at a dose of 8 Gy. Repeat the material stimulation and radiation therapy after three days. The weight and tumor size of mice were measured every other day. The experiment was terminated after 25 days of treatment, and the mice were sacrificed using carbon dioxide, and the tumor tissues were stored in 4% paraformaldehyde.

## Tissue staining

The tissue was embedded and sectioned after fixation and gradient alcohol dehydration. Next, the sections were dewaxed and antigen repaired. For immunohistochemical staining, follow the instructions (KIT-9706, MXB Biotechnologies). Peroxidase blocking, serum blocking, antibody incubation (IL-1β, 1:200, P50520-1R1, Abmart; IL-18, 1:200, P50519-2R2, Abmart; CRT, 1:200, 27298-1-AP, Proteintech; HSP90β, 1:400, A23489, Abclonal), secondary antibody labeling and other steps were carried out in turn, and DAB staining solution(ZLI-9017, ZSGB-BIO) was used for color development. For immunofluorescence staining, serum was used to block for one hour and antibody (Ki67, 1:200, ab16667, abcam) was added to incubate overnight at four degrees. The next day, the fluorescent secondary antibody (1:200, SA00013-4, Proteintech) was used for incubation, and the tablets were sealed with Antifade Mounting Medium with DAPI (P0131, Beyotime). For TUNEL staining, follow the instructions (C1086, Beyotime). Proteinase K (ST533, Beyotime) was used to incubate for 30 min at room temperature, and then TUNEL working solution was added to incubate for 1 h at 37 ℃. The tablets were sealed with Antifade Mounting Medium with DAPI (P0131, Beyotime).

## Oxy-Hemo photoacoustic (PA) imaging

Oxy-Hemo photoacoustic images and oxygen saturation (sO_2_) average total (%) of the tumor and peritumoral tissue were obtained using Vevo 3100 LAZR system (FUJIFILM VisualSonics, Inc.). Oxygen saturation (sO_2_) is calculated as the percentage of oxygenated hemoglobin relative to total hemoglobin based on dual-wavelength PA imaging at 750 nm/850 nm.

## Live imaging of small animals

Inject 6 × 10^6^ CAL 27 tumor cells into the root of tongue in mice and perform in vivo imaging 21 days later. Inject 0.3 mg nanoparticles into mice through the tail vein. 4 hours later, the tissues were imaged using IVIS SPECTRUM for measuring the biodistribution of nanoparticles.

## Hemolysis test

The nanomaterials with different weights were placed in 1ml PBS, and 200 μL of defibrillated sheep blood was added. The negative control did not add material, and the positive control was 1 mL ACK erythrocyte lysate (C3702, Beyotime). Incubate at 37 ° C for 30 min and centrifuge at 2500 rpm for 5 minutes. The supernatant was taken for absorbance test (OD 450nm). The hemolysis rate of each group is calculated according to the following formula:

$$Hemolysis rate=\frac{Abs(experiment)}{Abs(lysis buffer)}\times100\%$$

## Biosafety Testing

Twelve six-week-old female Balb/c nude mice were used for the experiment. Different groups of nanoparticles were injected through the tail vein. The mice were sampled after 14 days. Blood was taken under anesthesia. One part of blood was tested by routine blood test, and the other part of serum was harvested for biochemical index test. At the same time, the main organs (heart, liver, spleen, lung and kidney) were removed for tissue H&E staining.

## Statistical analysis

Unless otherwise specified, data are expressed as the mean ± SD. All experiments were repeated at least three times. The comparison between the two groups was performed using an unpaired two-tailed t-test. For comparison between three or more independent groups, one-way analysis of variance (ANOVA) was performed using Tukey's multiple comparisons test. Statistical analysis was performed using GraphPad Prism 7. When P < 0.05, the difference was considered statistically significant.

## Data available

All the primary data supporting the results of this study are provided in the paper and its supplementary information. RNA transcriptome sequencing data and other data that support this article can be obtained from the corresponding author.

# Supplementary figures


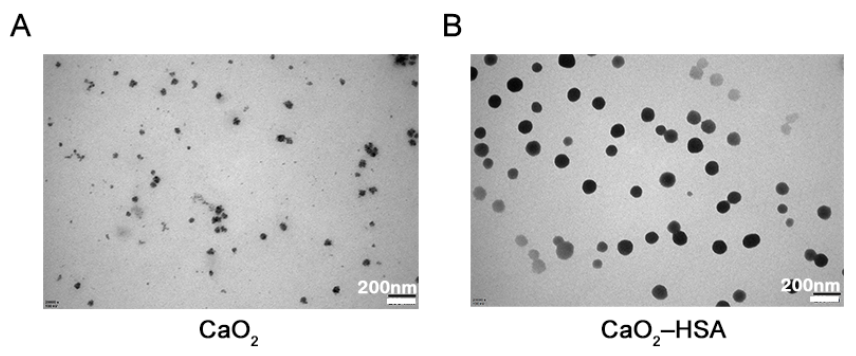


**Figure S1.** TEM image of CaO_2_ (A) and CaO_2_-HSA (B).


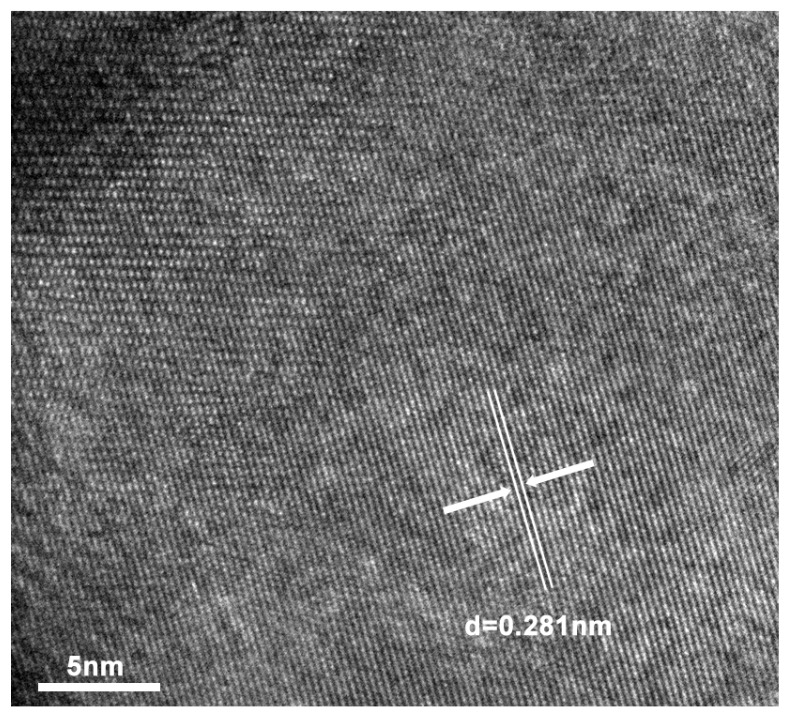


**Figure S2.** High-resolution TEM image of the CaO_2_-HSA nanocrystals.


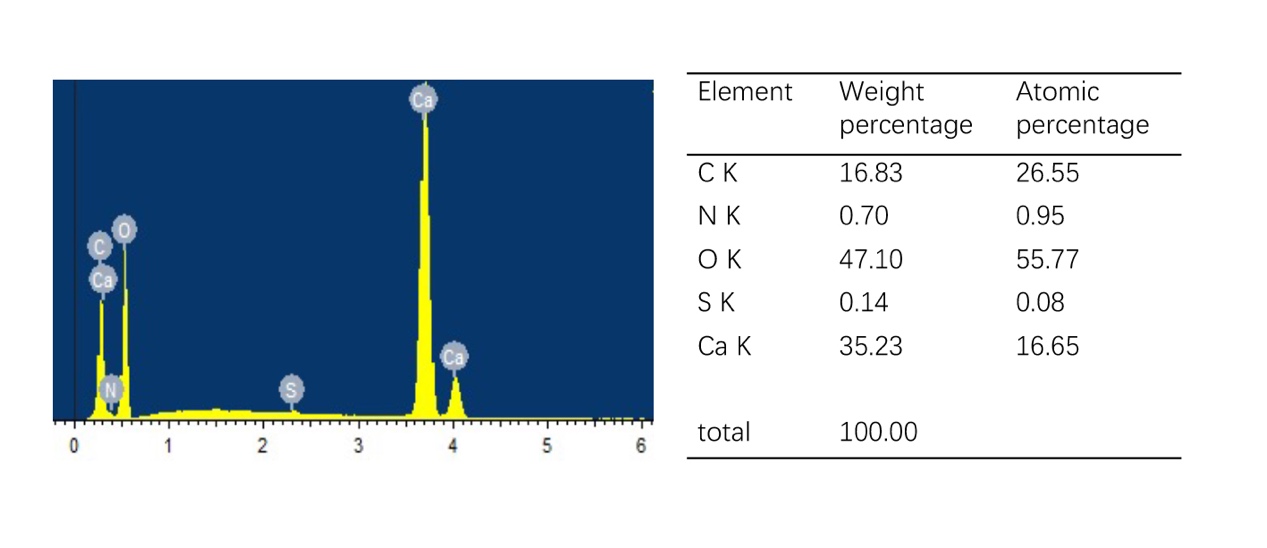


**Figure S3.** Scanning electron microscopy EDS analysis of CaO_2_-HSA nanomaterial.


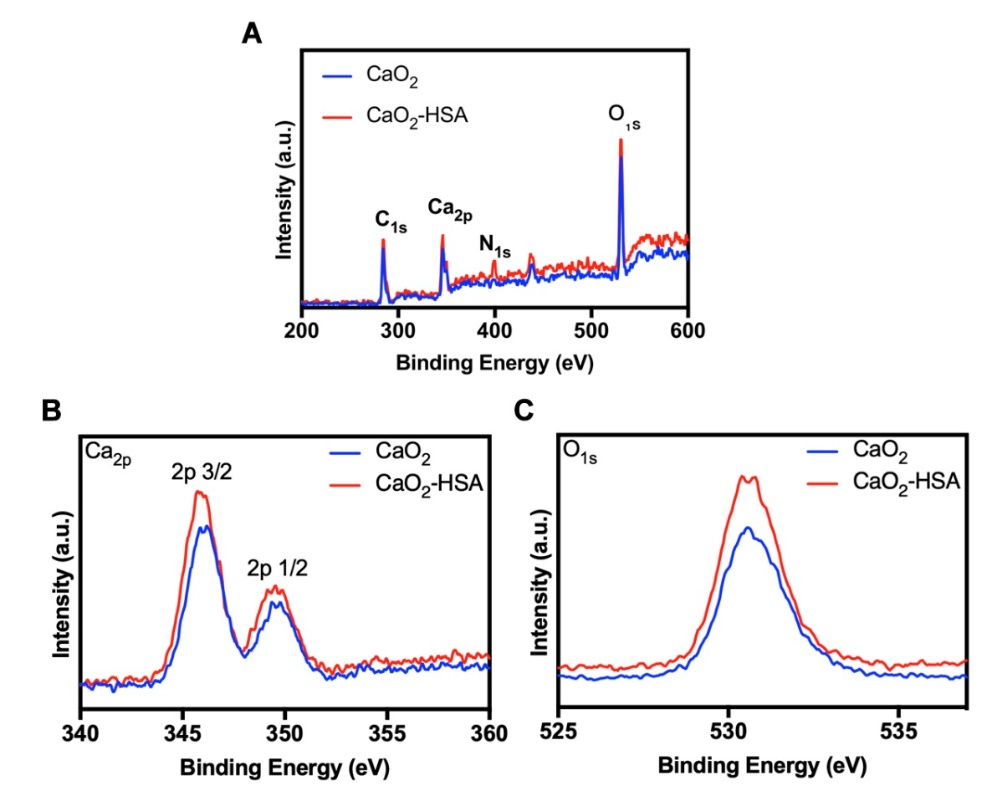


**Figure S4.** (A) XPS spectra of the CaO_2_ and CaO_2_-HSA nanoparticles; XPS high-resolution spectrum of Ca 2p and O1s are shown in (B) and (C), respectively. The XPS results showed that CaO_2_-HSA is composed of Ca, O, N, and C.


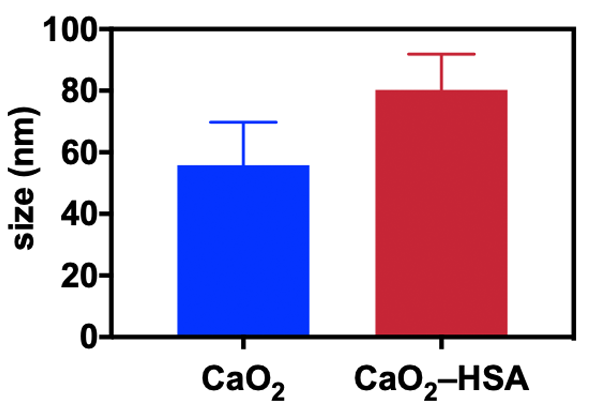


**Figure S5.** DLS measurements of hydrodynamic size (diameter) of CaO_2_ and CaO_2_-HSA. Data presented as mean ± s.d. (n = 3).

**
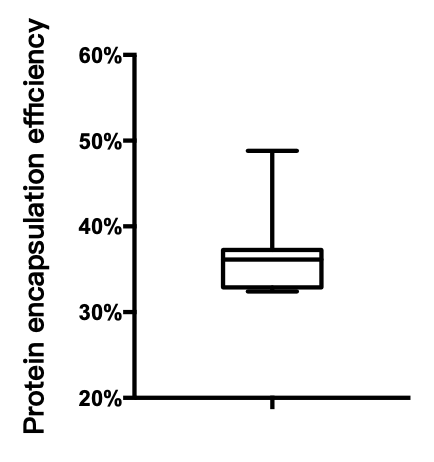
**

**Figure S6.** The encapsulation efficiency of HSA in CaO_2_-HSA nanomaterial was tested by Bradford method. Data presented as mean ± s.d. (n = 13).


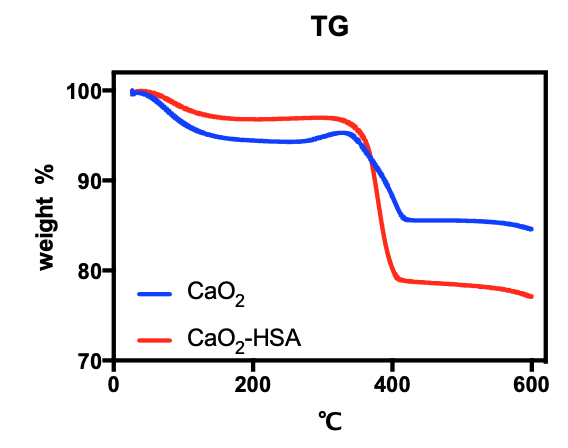


**Figure S7.** Thermogravimetric analysis of CaO_2_ and CaO_2_-HSA.

**
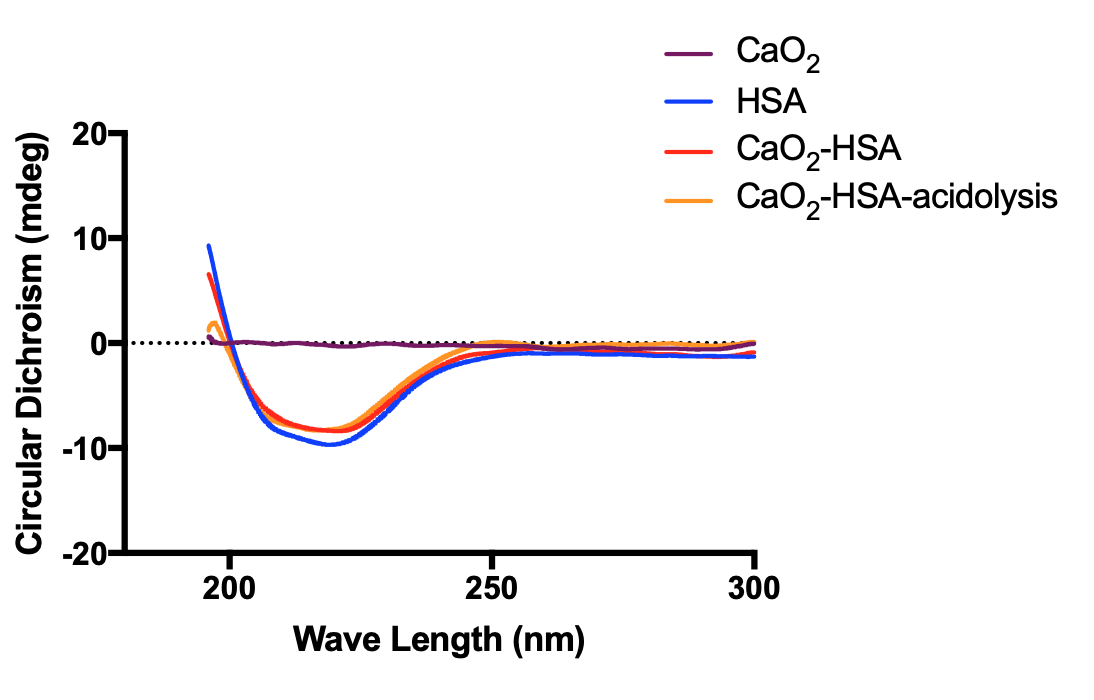
**

**Figure S8.** Circular dichroism shows the change in secondary structures of HSA. Using the same concentration of HSA as the standard, the secondary structures of CaO_2_-HSA and acid treated CaO_2_-HSA supernatant (released HSA) were not significantly affected.


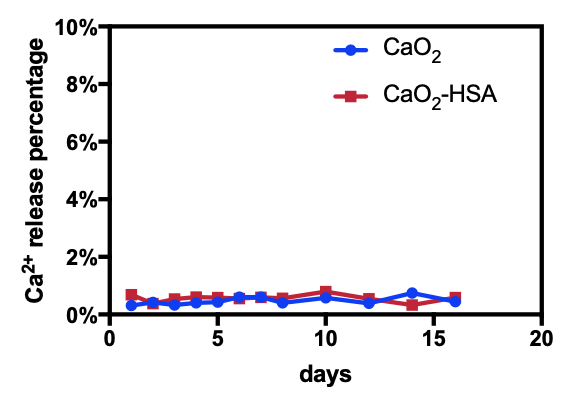


**Figure S9.** The long-term stability of nanoparticles in PBS (pH = 7.4) was studied by measuring amount of calcium ions released in the supernatant.


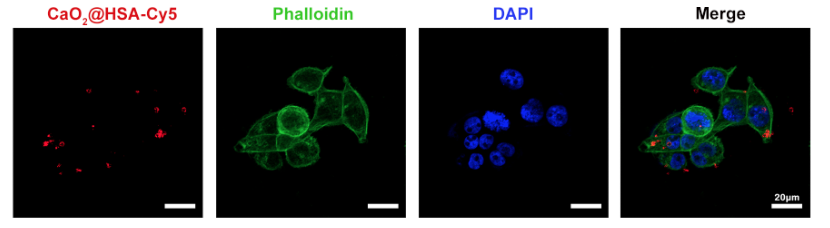


**Figure S10.** Fluorescence confocal microscopy images show the entry of nanomaterials into CAL 27 cells. Red fluorescence represents CaO_2_-HSA, green fluorescence represents Phallodin (cytoskeleton), and blue fluorescence represents DAPI (Cell nuclei).


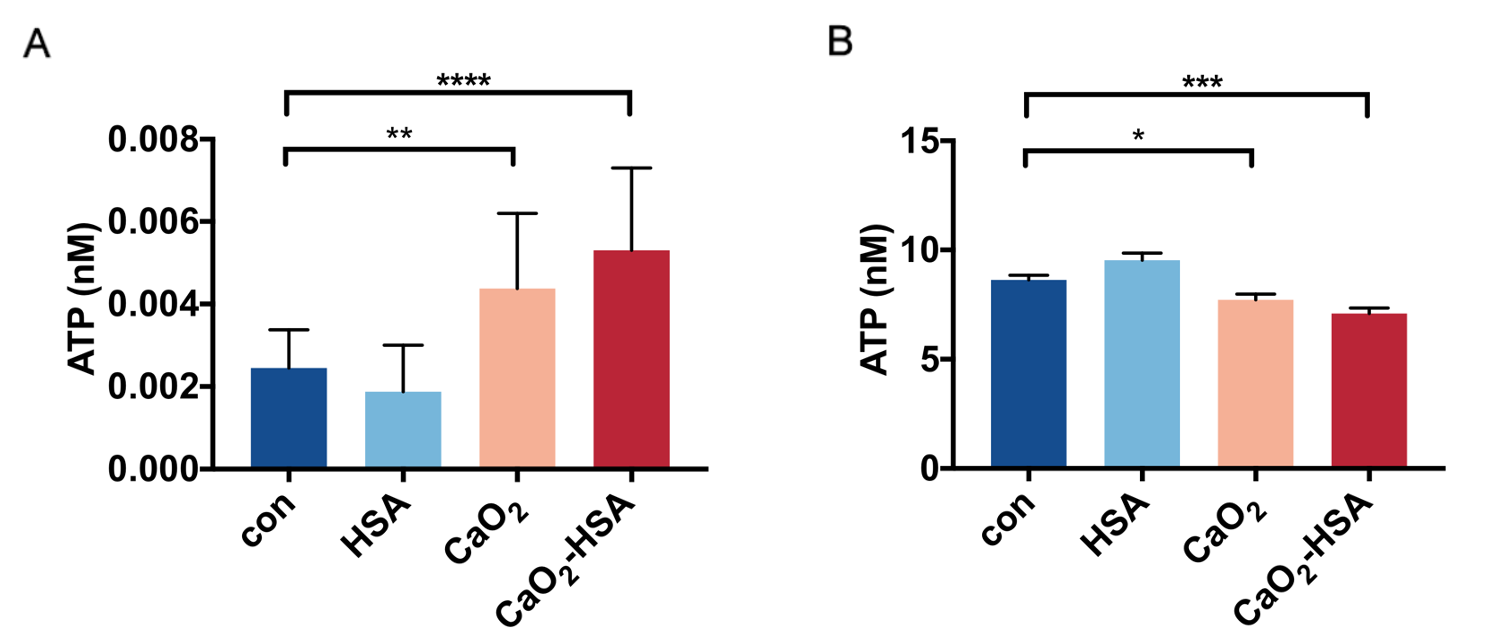


**Figure S11.** the ATP content both in the cellular supernatant (A) and within the cells (B) after adding different groups of materials were detected. Data presented as mean ± s.d. (n = 3~5) (* means p < 0.05, ** means p < 0.01, *** means p < 0.001, **** means p < 0.0001).


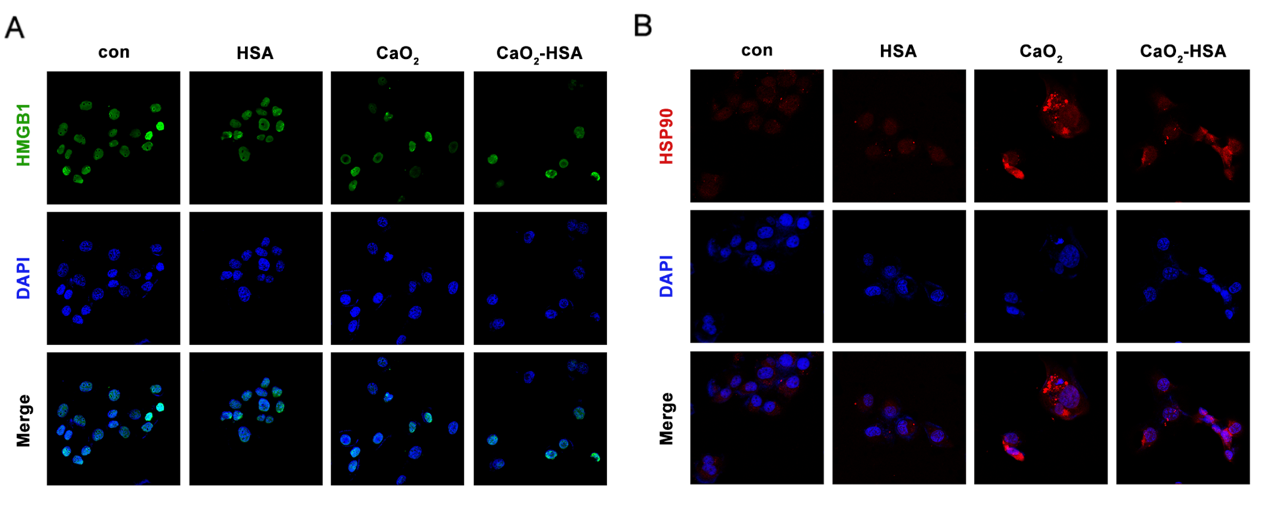


**Figure S12.** Confocal fluorescence showed the expression of HMGB1 and HSP90 in CAL 27 cells.


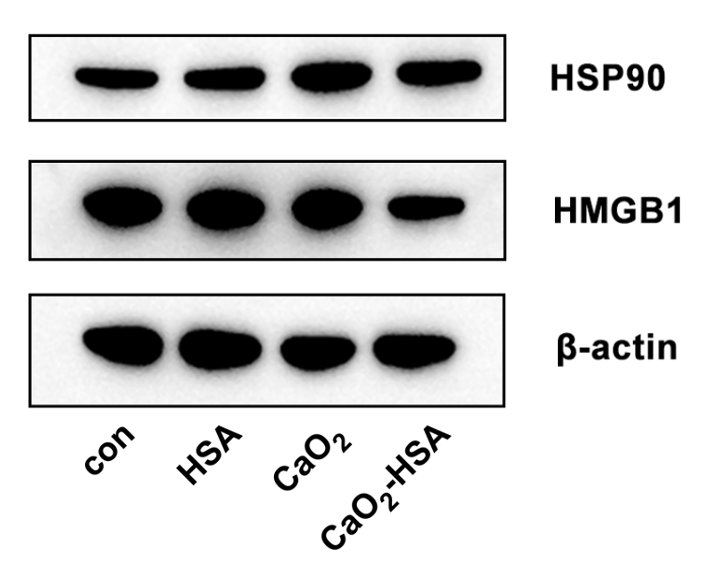


**Figure S13.** Western blot images of protein expression levels of HMGB1 and HSP90 in CAL 27 cells after stimulation of various materials.


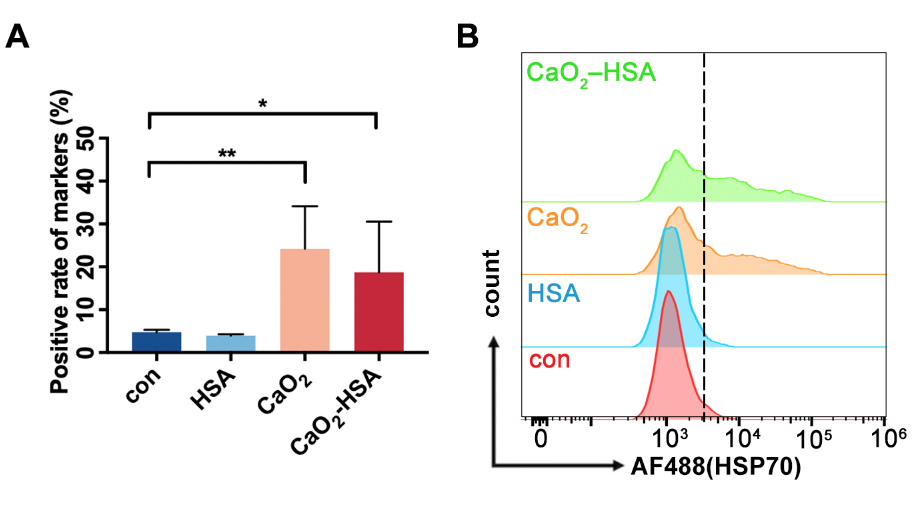


**Figure S14.** Expression of HSP70 in CAL27 cells after adding different groups of materials was detected by flow cytometry. Data presented as mean ± s.d. (n = 6) (* means p < 0.05, ** means p < 0.01).


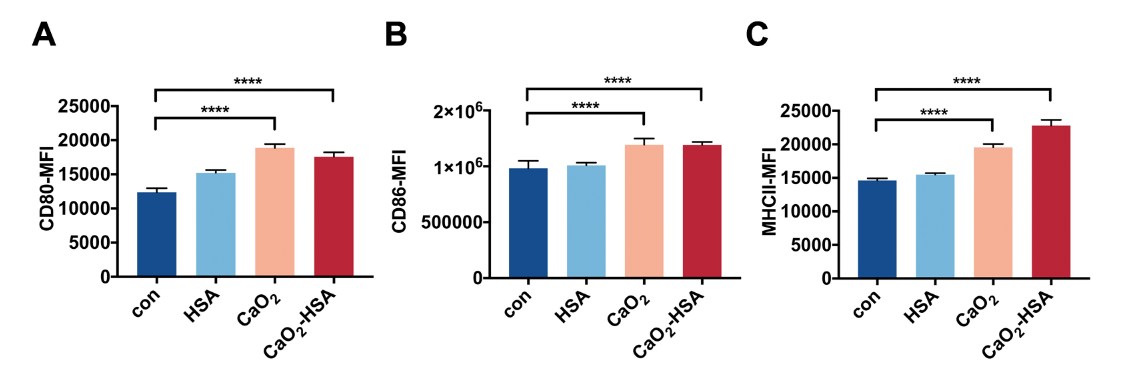


**Figure S15.** Expression of CD80, CD86 and MHC II in Dc 2.4 cells were detected by flow cytometry after adding different groups of cellular supernatants. Data presented as mean ± s.d. (n = 6) (**** means p < 0.0001).

**
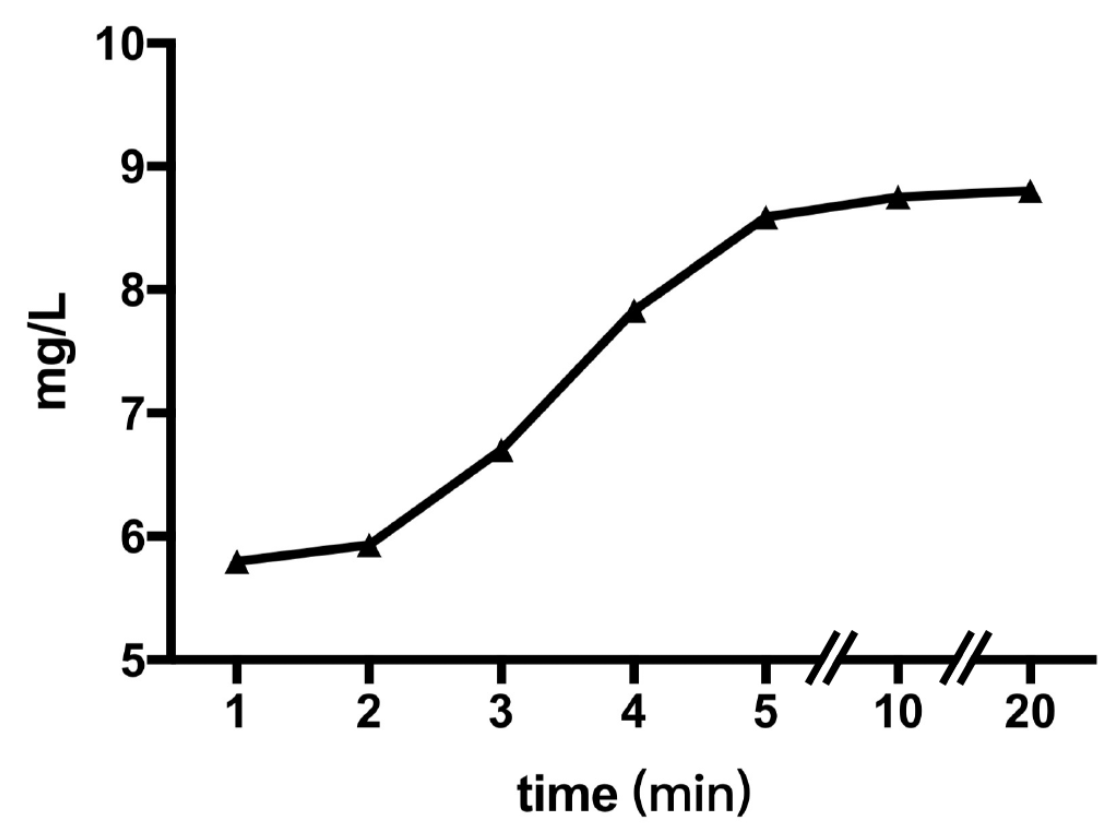
**

**Figure S16.** Dissolved oxygen content curve measured by placing CaO_2_-HSA in complete culture medium.


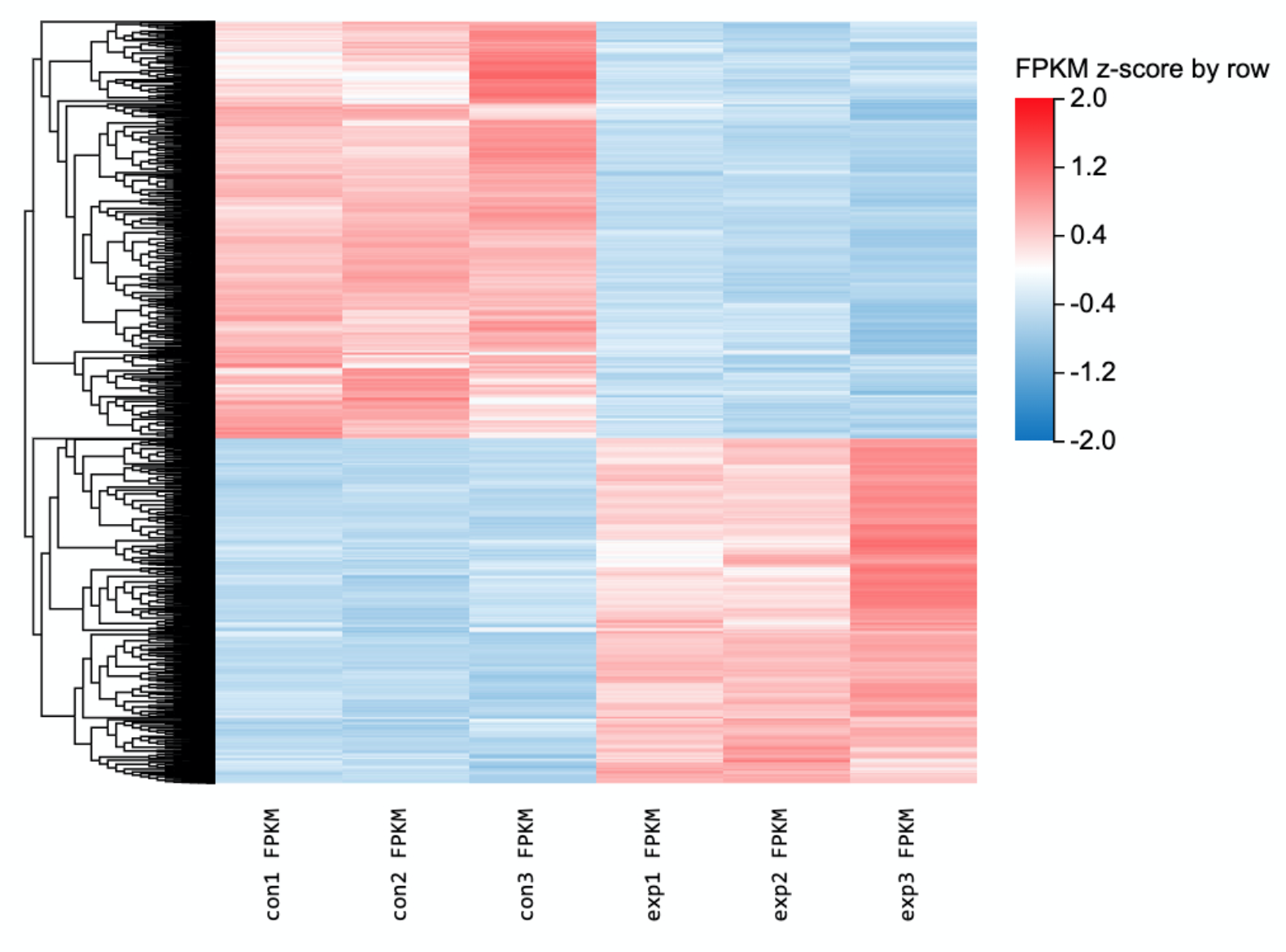


**Figure S17.** The heatmap showed the differentially expressed genes in the control (PBS) and experimental (CaO_2_-HSA) groups.


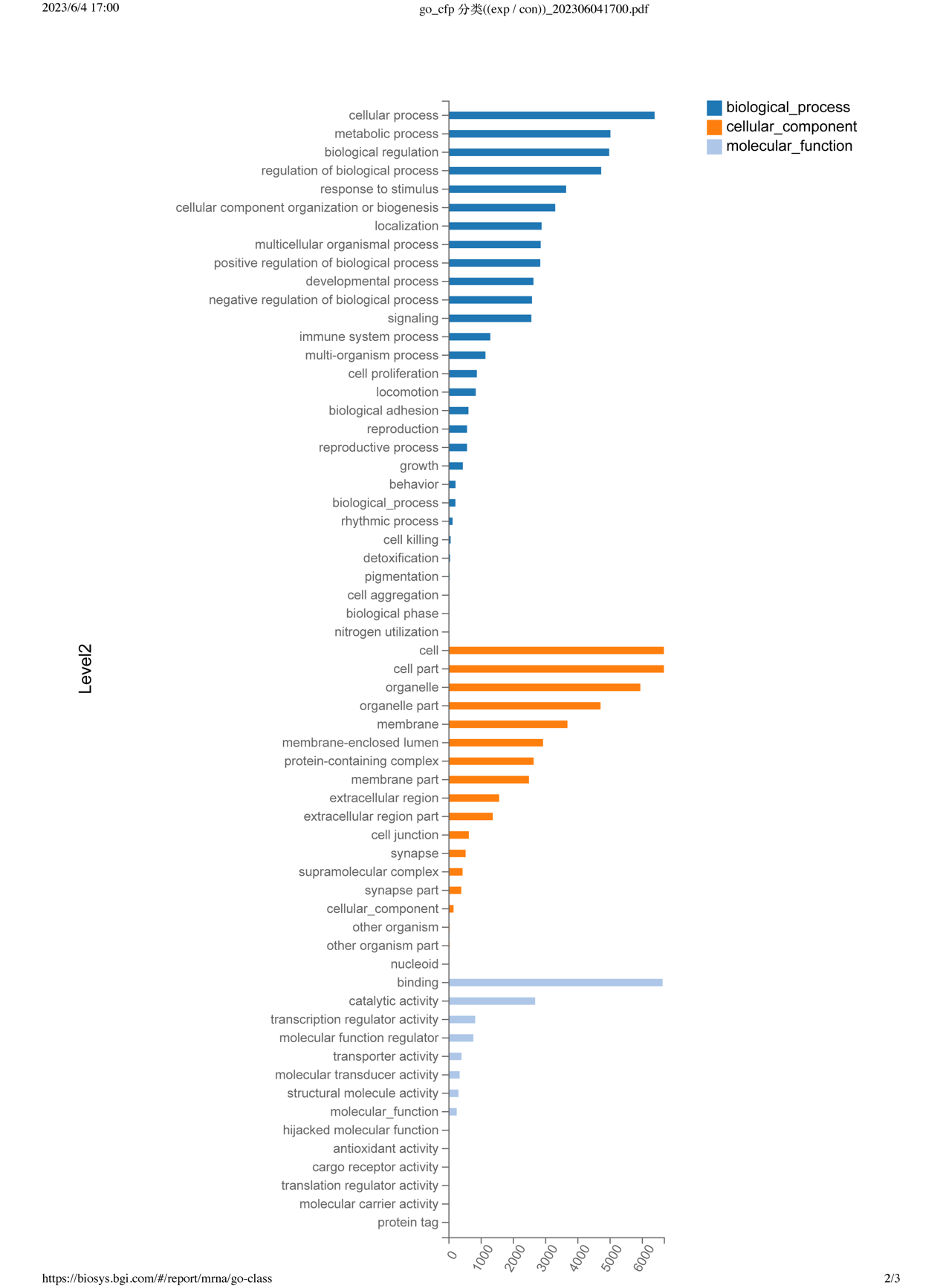


**Figure S18.** GO analysis was performed on all samples, showing a summary histogram for each classification.

**Figure S19.** KEGG pathway analysis was performed on all samples, showing a summary histogram for each classification.


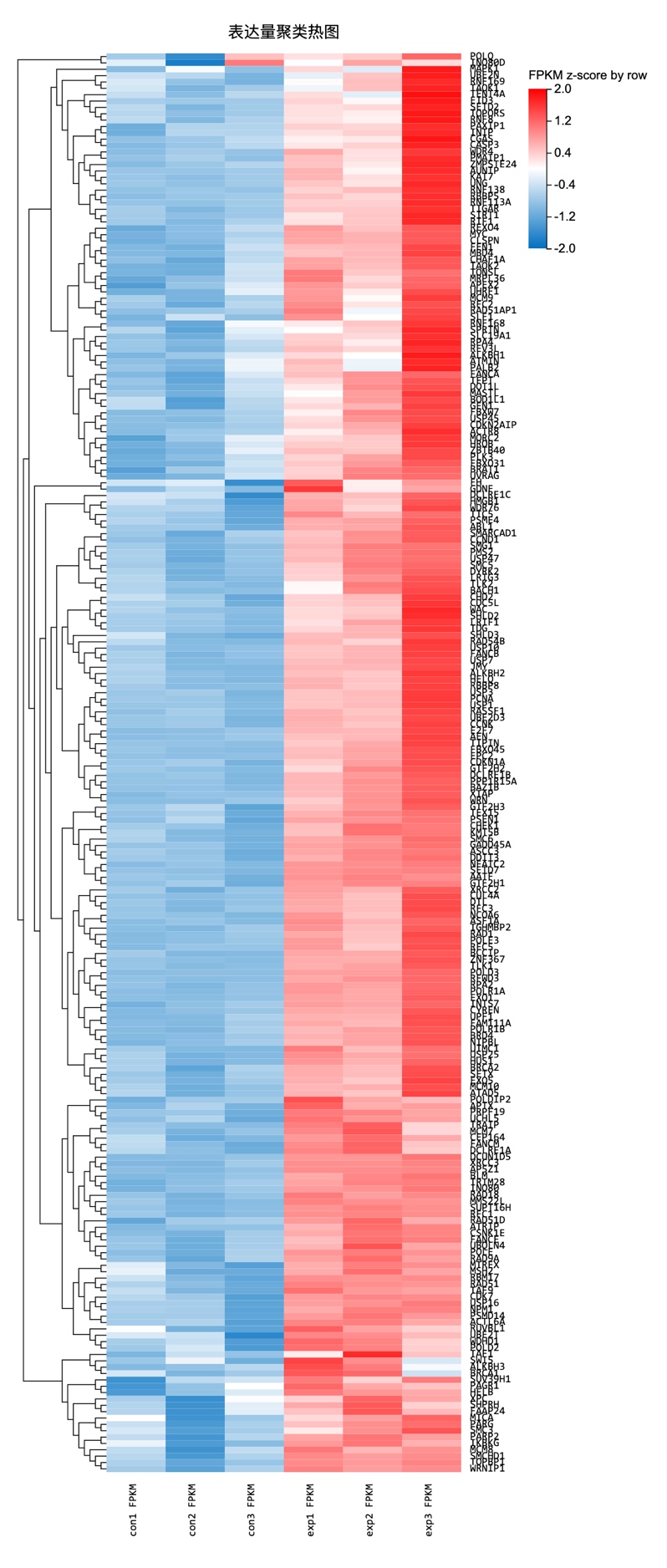


**Figure S20.** Heatmap of gene expression related to DNA damage and repair.

**
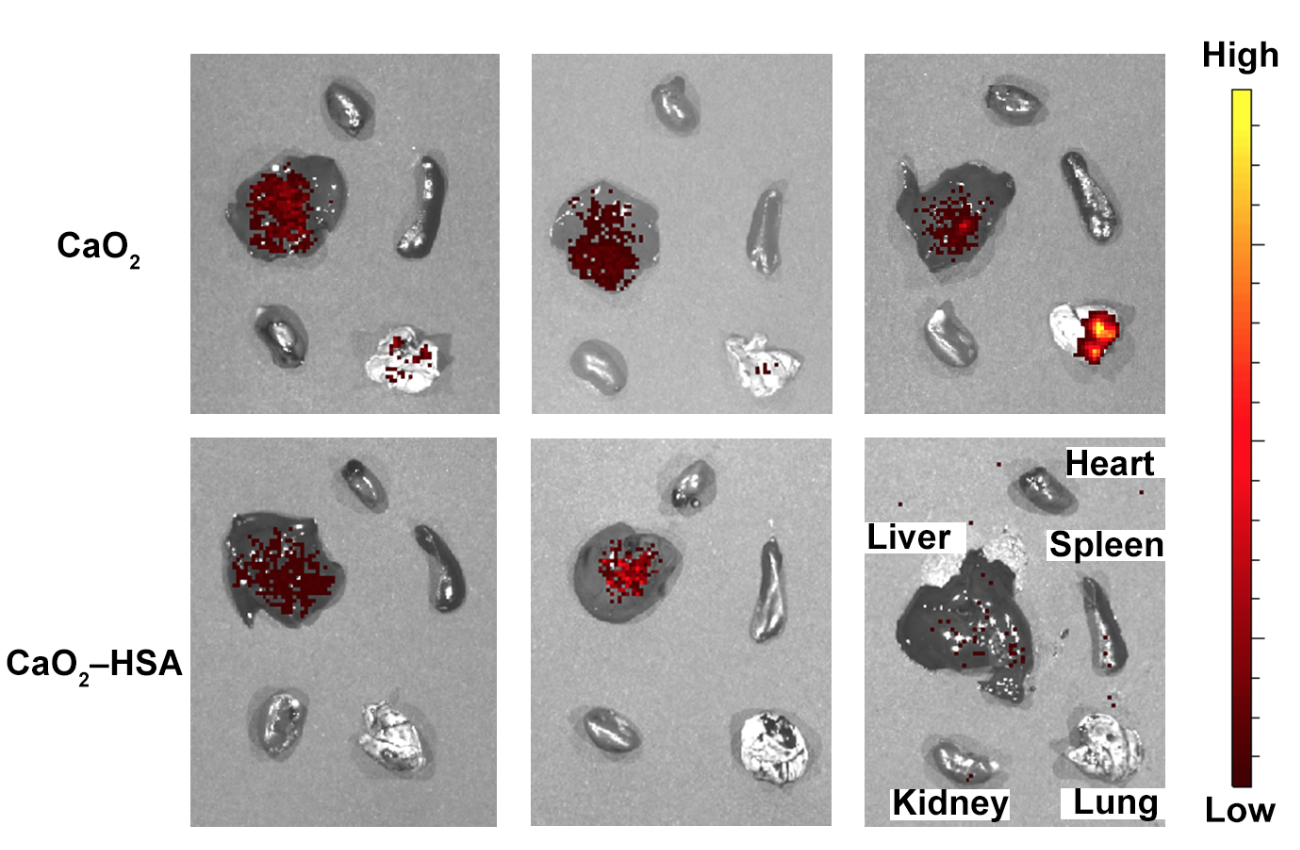
**

**Figure S21.** Fluorescence imaging of major organs in mice after 12 hours of drug injection into the tail vein.


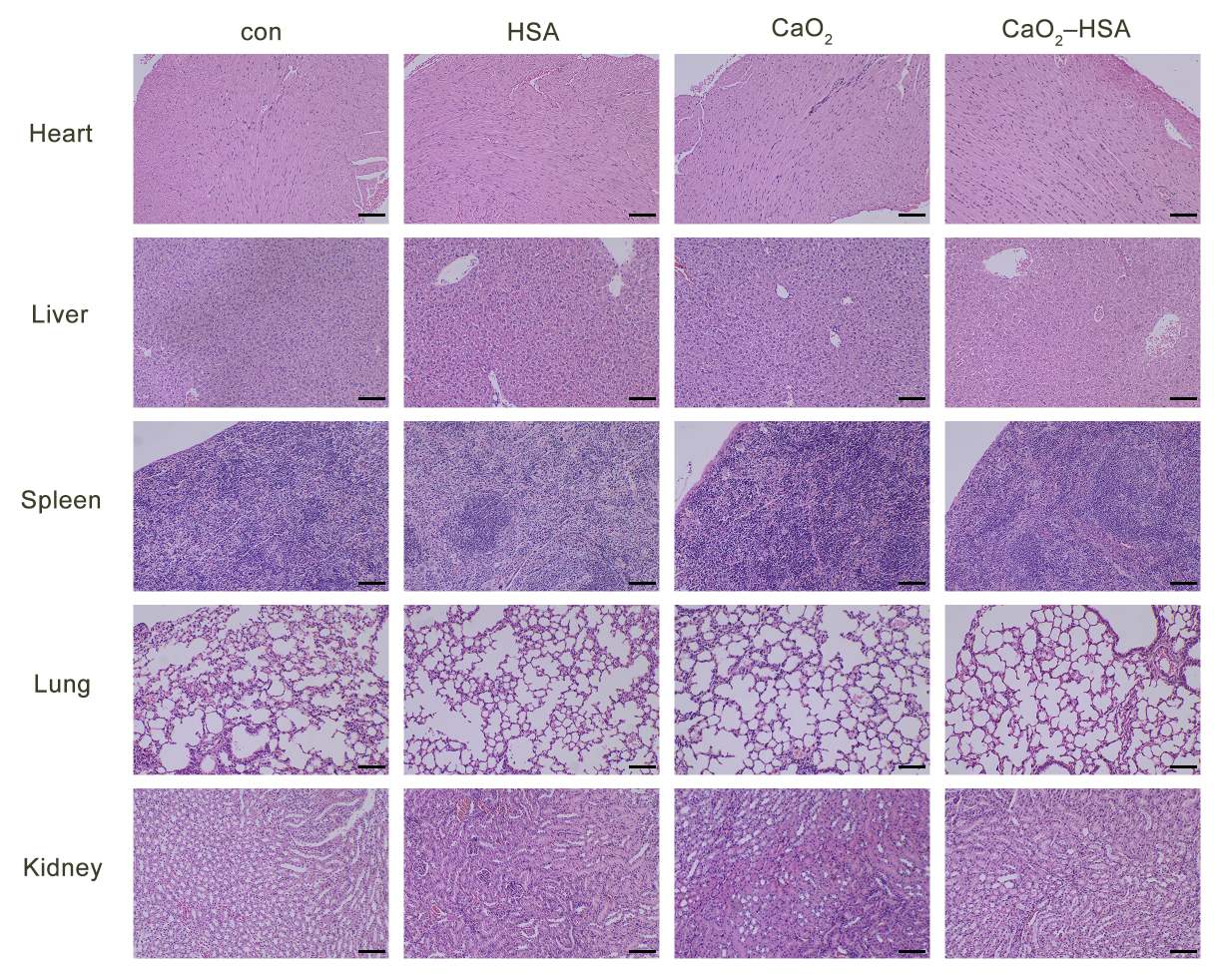


**Figure S22.** H&E staining of the main organs (heart, liver, spleen, lung and kidney) were used to evaluate the biosafety of nanoparticles in normal mice. Scale bars: 100 μm.


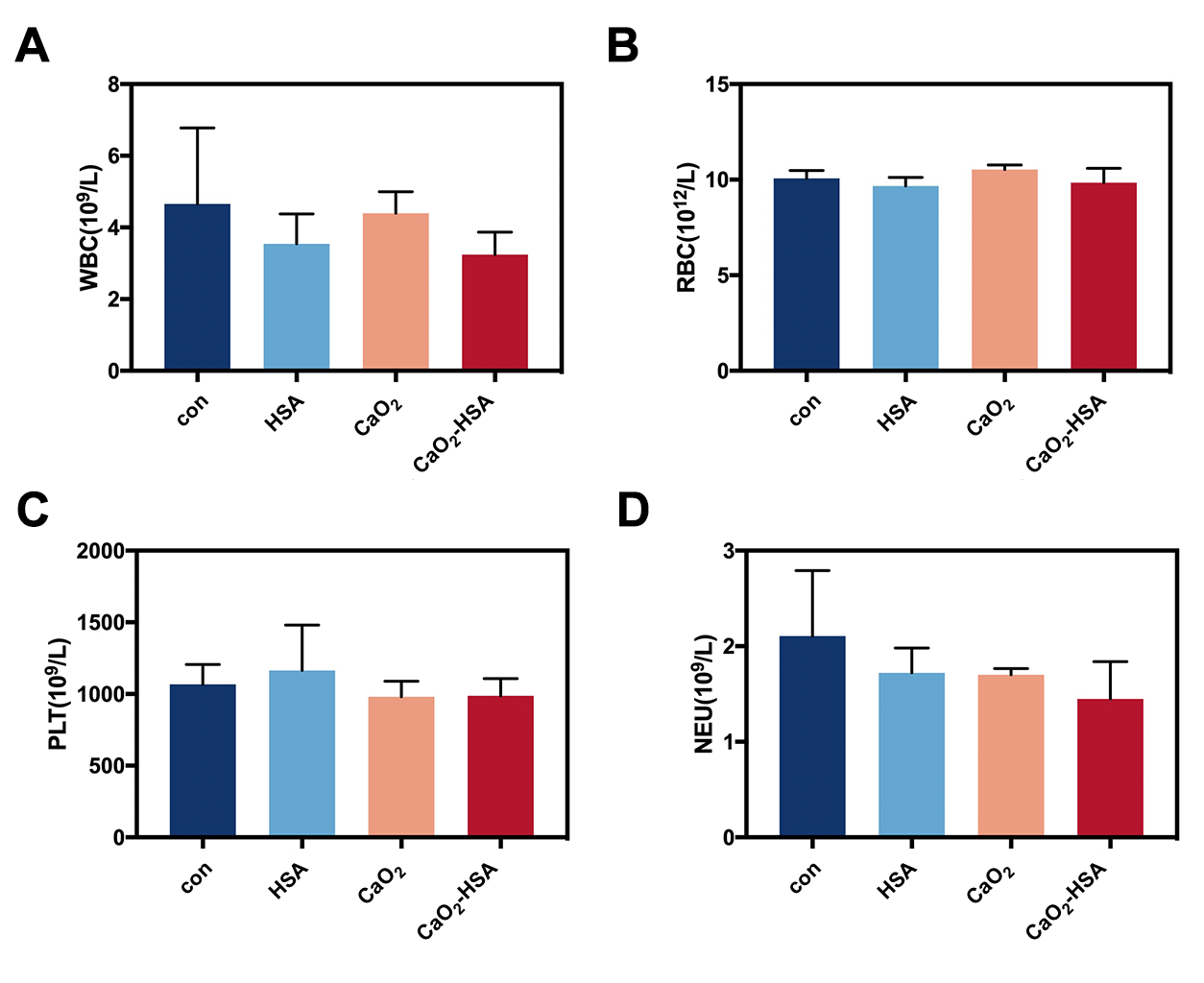


**Figure S23.** Hematological parameters of healthy mice after infusion of nanomaterials into tail vein (n = 3). There is no significant difference in each indicator between the groups (p > 0.05).


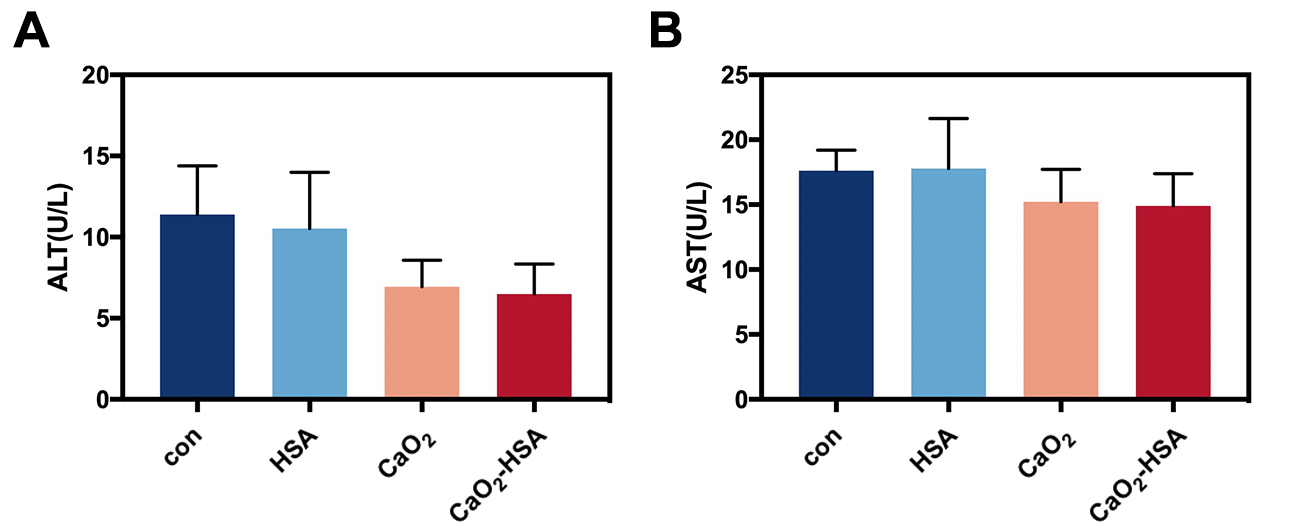


**Figure S24.** Blood biochemical parameters were detected after infusion of nanomaterials into the tail vein of healthy mice (n = 3). The nanomaterials did not cause significant increase of ALT and AST indices.


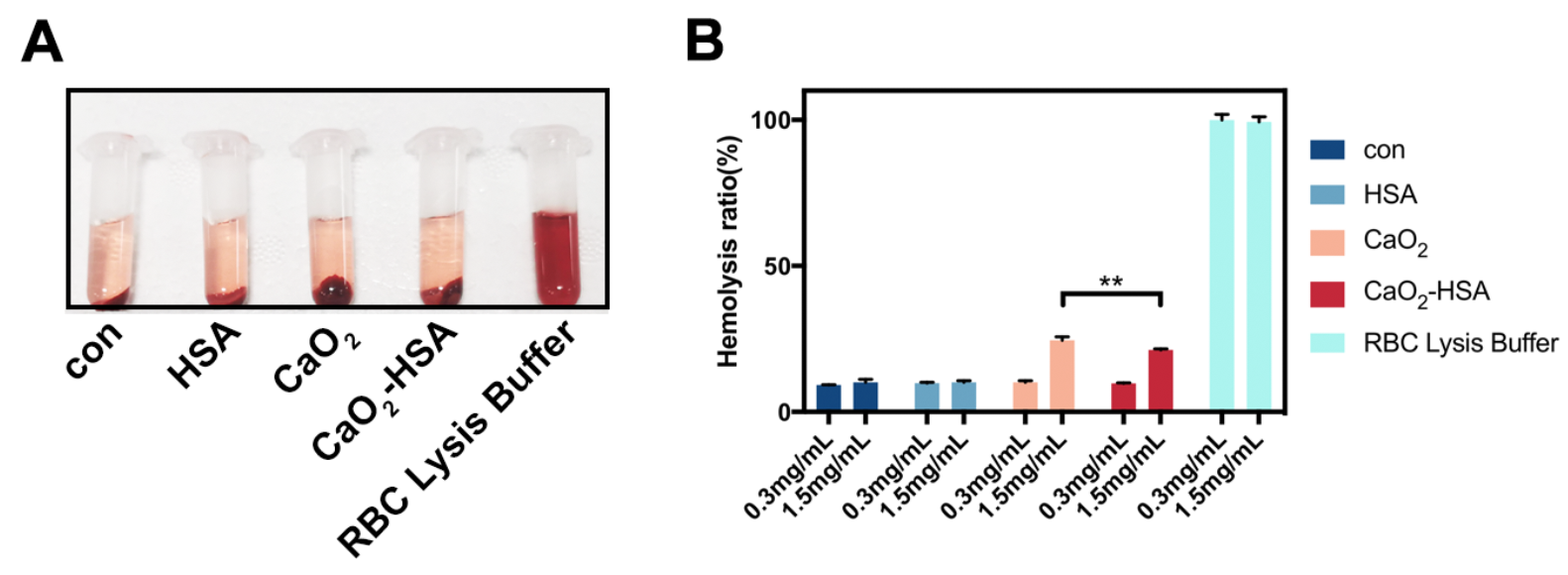


**Figure S25.** Photographs of the hemolytic capacity of the nanomaterials and the relative quantitative statistical chart. The added dose of 1.5mg/ml far exceeds the therapeutic dose (** means p < 0.01) .


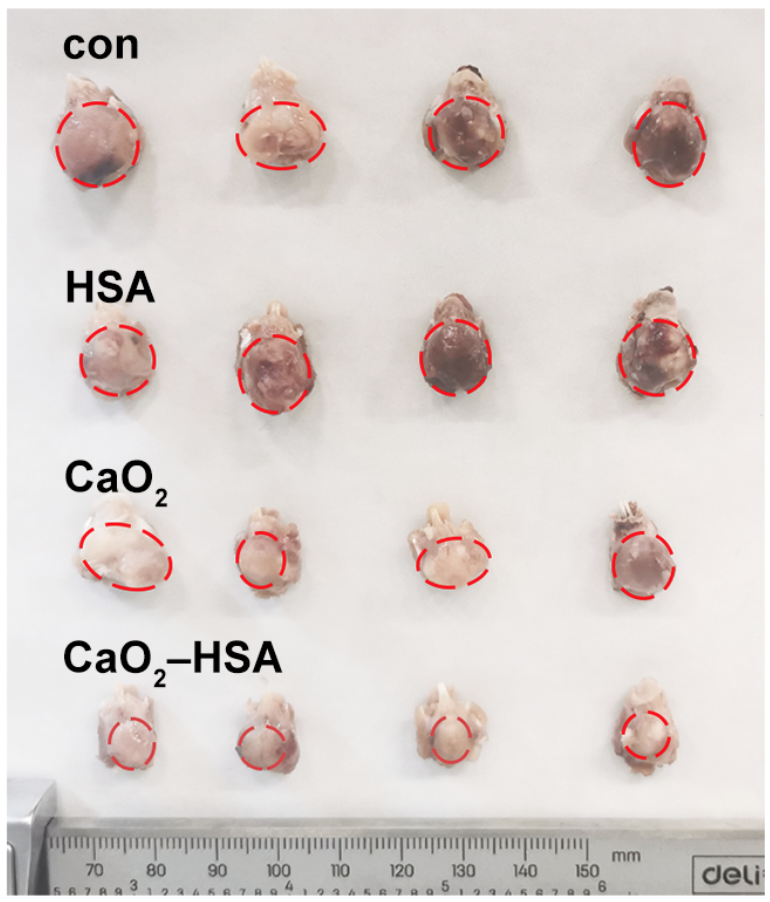


**Figure S26.** Photo of local tumor areas.

**
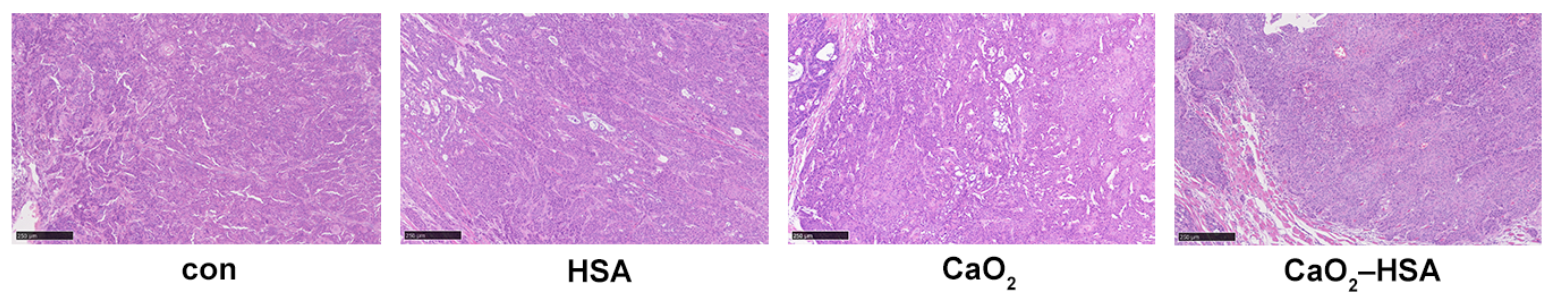
**

**Figure S27.** H&E staining images of tumor site. Scale bars: 100 μm.


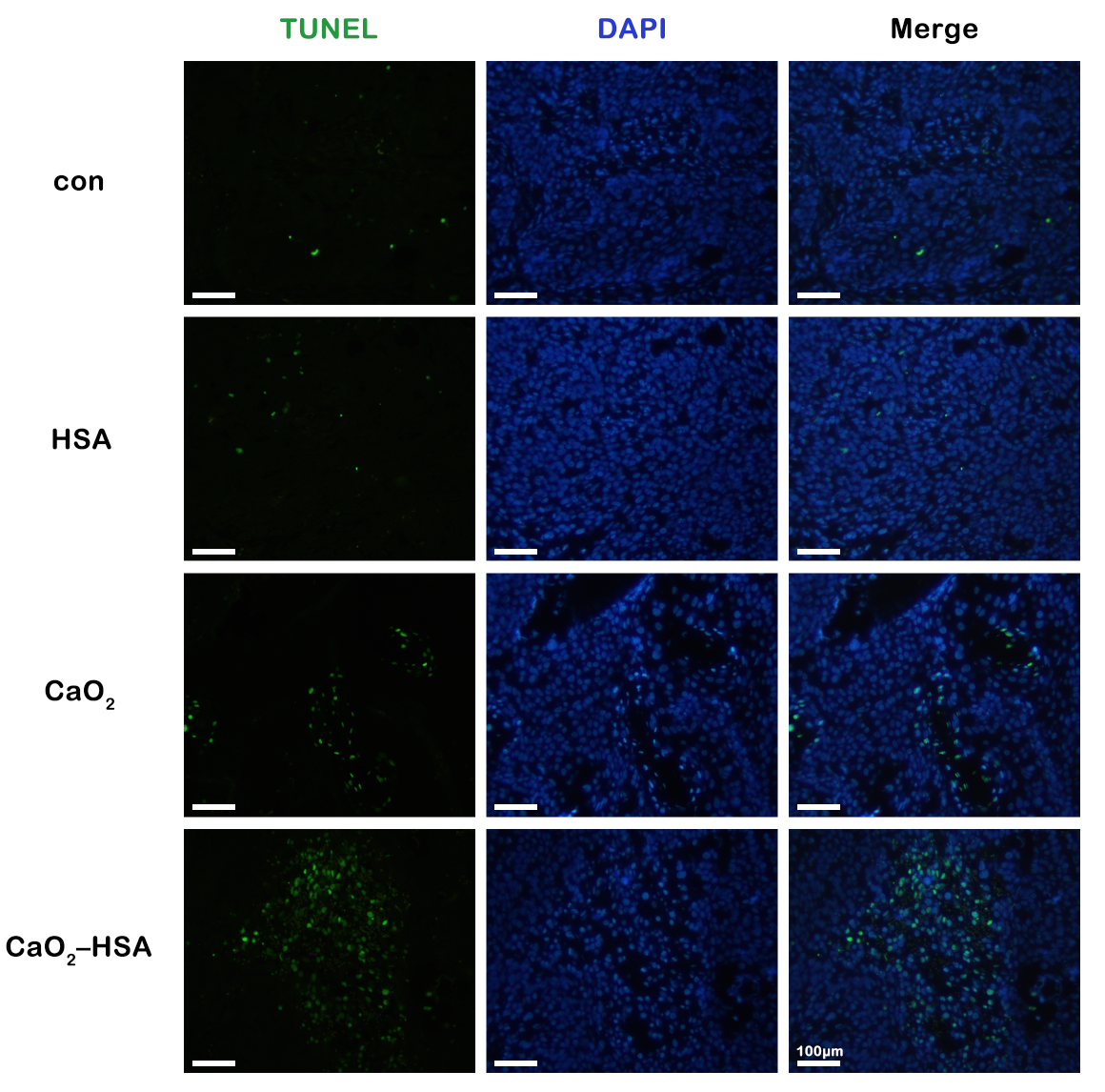


**Figure S28.** The TUNEL index of tumor sites in different groups of mice were evaluated by immunofluorescent staining. (Scale bars: 100 μm)


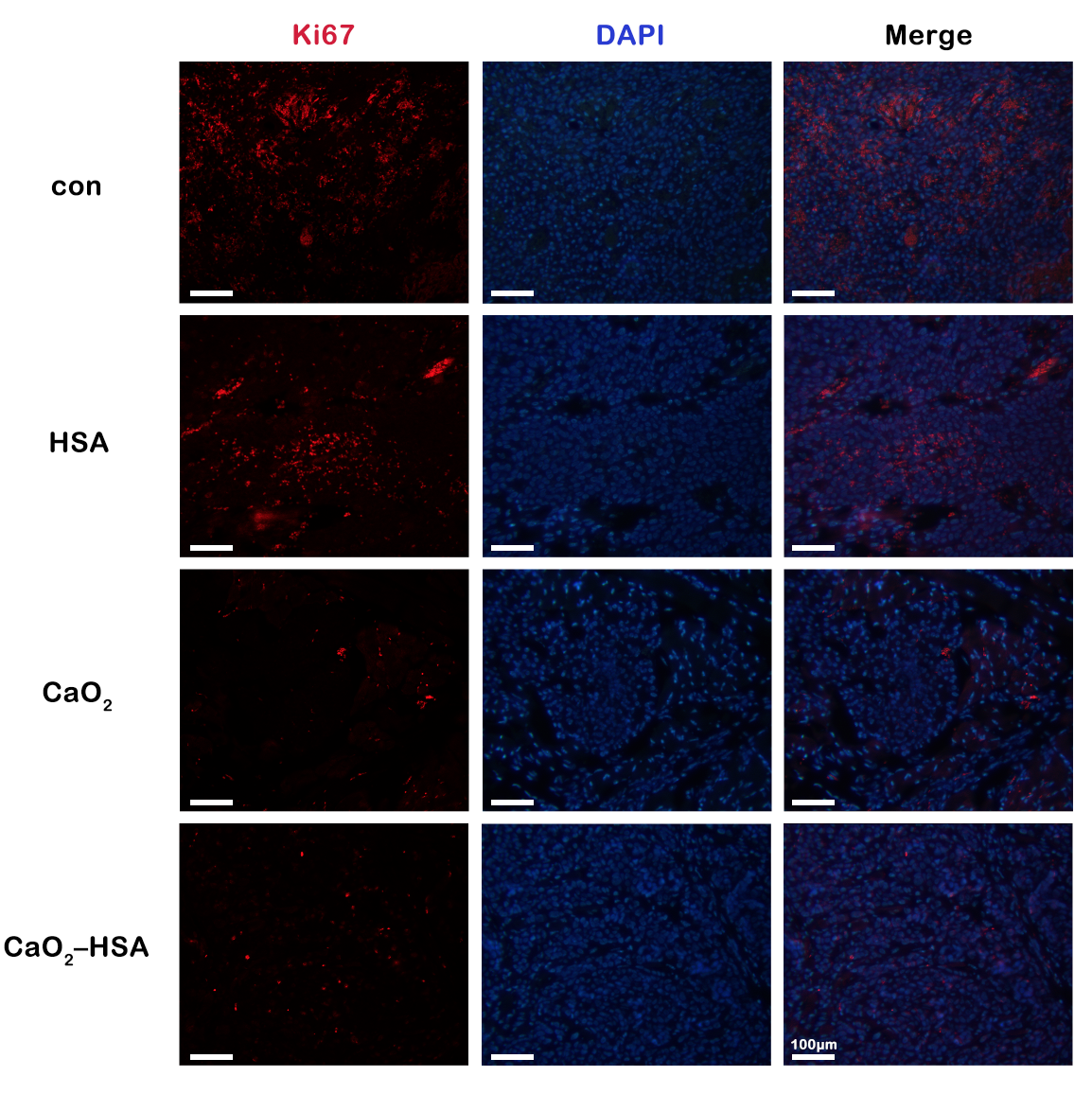


**Figure S29.** The Ki67 index of tumor sites in different groups of mice were evaluated by immunofluorescent staining. (Scale bars: 100 μm)


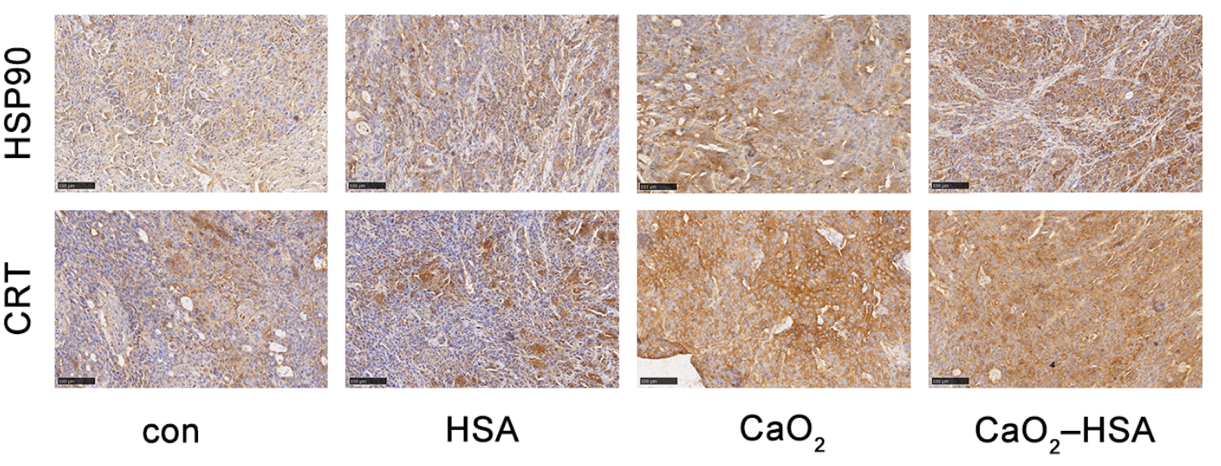


**Figure S30.** The expression of HSP90 and CRT in tumor sites of mice in different groups were evaluated by immunohistochemical staining. (Scale bars: 100 μm)


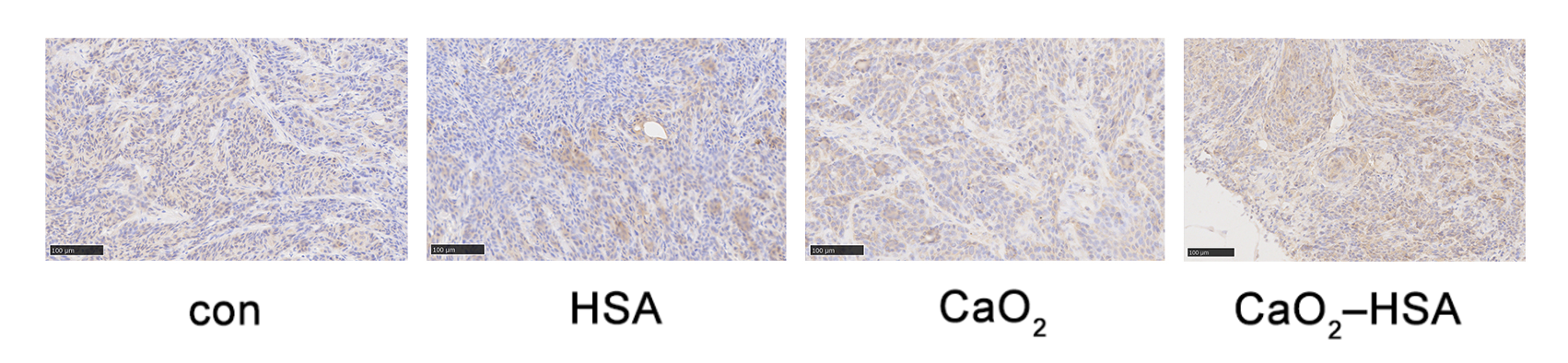


**Figure S31.** The expression of IL18 in tumor sites of mice in different groups were evaluated by immunohistochemical staining. The larger the brown range and darker the color inside the cell indicate the higher the expression level of IL-18. (Scale bars: 100 μm)

**Table S2.** Details of the common radiosensitizers on the market/in research.

| **Material name** | **SER value** | **Material type** | **Mechanism** | **Reference** |
| --- | --- | --- | --- | --- |
| topotecan | 1.16 | Small molecule | inhibit activity of topoisomerase I helps stop DNA damage repair and increase DNA double-strand breaks lethal damages | ^[1]^ |
| nelfinavir | 1.2 | Small molecule | inhibition of the PI3K-AKT-mTOR pathway | ^[2]^ |
| Piper longumine | 1.22 | Small molecule | regulation of apoptosis-related protein expression and the increase of intracellular ROS level, thus increasing radiation-induced apoptosis | ^[3]^ |
| docetaxel-loaded nanoparticles (DOC-NPs) | 1.24 | Other nanomaterial | enhanced G2/M arrest, increased reactive oxygen species (ROS), more effective DNA double-strand breaks and promoted apoptosis | ^[4]^ |
| doranidazole | 1.3 | Small molecule | suppression of endogenous radio-protective substances, cytotoxic compounds production by radiolysis, inhibition of cellular repair, structural incorporation of thymine into intracellular DNA, and oxygen-mimicking substances | ^[5]^ |
| sodium glycididazole | 1.32 | Small molecule | selective reaction with DNA radicals | ^[6]^ |
| anti-EGFR-iRGD-conjugated (iE)-PRNPs | 1.32 | Other nanomaterial | enhanced G2/M arrest, increased reactive oxygen species, and more effective induction of DNA double-strand breaks | ^[7]^ |
| AZD-1390 | 1.36 | Small molecule | inhibit the key DNA damage response proteins (ATM) | ^[8]^ |
| A-966492 | 1.39 | Small molecule | inhibit activity of PARP helps stop DNA damage repair and increase DNA double-strand breaks lethal damages | ^[1]^ |
| albumin-modified GNPs | 1.432 | Other nanomaterial | enhance the absorption cross-section of X-ray, improve the relative dose accumulation of tumors, promote the generation of free radicals, and enhance the ability of DNA damage | ^[9]^ |
| nimorazole | 1.45 | Small molecule | fixing radiation-induced damage under hypoxic conditions as a substitute of oxygen | ^[10]^ |
| hTR ASODN | 1.479 | Macromolecule | enhance the anti‑tumor effects of radiation by inducing cell apoptosis | ^[11]^ |
| nimotuzumab | 1.5 | Macromolecule | inhibition of EGFR signalling | ^[12]^ |
| Au NCs@His | 1.54 | High-Z nanomaterial | decrease the intracellular GSH level, thus preventing the ROS from being consumed by GSH，arrest the cells at the radiosensitive G2/M phase | ^[13]^ |
| sanazole | 1.55 | Small molecule | fixing radiation-induced damage under hypoxic conditions as a substitute of oxygen | ^[10]^ |
| PSMA-AuNPs | 1.55 | High-Z nanomaterial | increasing the DNA damage in close proximity to the NPs due to the increased photoelectric interaction and increased Auger electron production | ^[14]^ |
| dextran-coated iron oxide (SPION-DX) nanoparticles | 1.61 | Other nanomaterial | enhancement of repairable or indirect damage to the cells,iron oxide catalyse ROS production in the cells | ^[15]^ |
| PEG-b-P(PLG-g-MN) | 1.62 | Other nanomaterial | reduce the repairing ability of the damaged cells | ^[16]^ |
| (PEG)-coated Ag@Au core-shell nanoparticles | 1.62 | High-Z nanomaterial | the nanoparticles with heavy metal atom in the tumor can concentrate the radiation energy within tumor tissue and increase the production efficiency of toxic reactive oxygen species. | ^[17]^ |
| AS1411/GNPs | 1.66 | Other nanomaterial | AS1411 aptamer has enhanced the radiation-induced cell death by increasing Au uptake | ^[18]^ |
| M3814 | 1.67 | Small molecule | inhibit the key DNA damage response proteins(DNA-PK) | ^[8]^ |
| superparamagnetic iron oxide nanoparticles (SPIONs) | 1.74 | Other nanomaterial | high surface-to-volume ratio, may act as a catalyst for the generation of ROS | ^[19]^ |
| AuNC-ASON | 1.81 | High-Z nanomaterial | exhibit a strong X-ray photon capture cross-section and high Compton scattering effect | ^[20]^ |
| AgNPs | 1.84 | High-Z nanomaterial | higher probability of emitting secondary radiation | ^[21]^ |
| KU-2285 | 1.95 | Small molecule | fixing radiation-induced damage under hypoxic conditions as a substitute of oxygen | ^[10]^ |
| MicroRNA-150 | 2.05 | Macromolecule | inhibition of the AKT pathway in NK/T cell lymphoma treatment | ^[22]^ |
| DOX@HMs | 2.18 | Macromolecule | higher electron affinity | ^[6]^ |
| CAC4A•AQ4N | 2.349 | Macromolecule | AQ4 is a DNA intercalator and potent inhibitor of DNA type II topoisomerase | ^[23]^ |

**References**

[1] F. Koosha, A. Neshasteh-Riz, A. Takavar, N. Eyvazzadeh, Z. Mazaheri, S. Eynali, M. Mousavi, *Biochem Biophys Res Commun* **2017**, *491*, 1092.

[2] J. Zeng, A. P. See, K. Aziz, S. Thiyagarajan, T. Salih, R. P. Gajula, M. Armour, J. Phallen, S. Terezakis, L. Kleinberg, K. Redmond, R. K. Hales, R. Salvatori, A. Quinones-Hinojosa, P. T. Tran, M. Lim, *Cancer Biol Ther* **2011**, *12*, 657.

[3] J. X. Yao, Z. F. Yao, Z. F. Li, Y. B. Liu, *Asian Pac J Cancer Prev* **2014**, *15*, 3211.

[4] F. B. Cui, R. T. Li, Q. Liu, P. Y. Wu, W. J. Hu, G. F. Yue, H. Ding, L. X. Yu, X. P. Qian, B. R. Liu, *Cancer Lett* **2014**, *346*, 53.

[5] R. Murata, M. Tsujitani, M. R. Horsman, *Radiother Oncol* **2008**, *87*, 331.

[6] W. Yin, M. Qiang, W. Ke, Y. Han, J. F. Mukerabigwi, Z. Ge, *Biomaterials* **2018**, *181*, 360.

[7] W. Ren, H. Sha, J. Yan, P. Wu, J. Yang, R. Li, H. Zhang, L. Yu, H. Qian, B. Liu, *J Biomater Appl* **2018**, *33*, 707.

[8] K. Bannik, B. Madas, S. Jarke, A. Sutter, G. Siemeister, C. Schatz, D. Mumberg, S. Zitzmann-Kolbe, *Sci Rep* **2021**, *11*, 23257.

[9] Y. Chen, S. Liu, Y. Liao, H. Yang, Z. Chen, Y. Hu, S. Fu, J. Wu, *Int J Nanomedicine* **2023**, *18*, 1949.

[10] C. Sugie, Y. Shibamoto, M. Ito, H. Ogino, H. Suzuki, Y. Uto, H. Nagasawa, H. Hori, *J Radiat Res* **2005**, *46*, 453.

[11] C. Yu, Y. Yu, Z. Xu, H. Li, D. Yang, M. Xiang, Y. Zuo, S. Li, Z. Chen, Z. Yu, *Mol Med Rep* **2015**, *11*, 2825.

[12] Y. Akashi, I. Okamoto, T. Iwasa, T. Yoshida, M. Suzuki, E. Hatashita, Y. Yamada, T. Satoh, M. Fukuoka, K. Ono, K. Nakagawa, *Br J Cancer* **2008**, *98*, 749.

[13] X. Zhang, X. Chen, Y. W. Jiang, N. Ma, L. Y. Xia, X. Cheng, H. R. Jia, P. Liu, N. Gu, Z. Chen, F. G. Wu, *ACS Appl Mater Interfaces* **2018**, *10*, 10601.

[14] R. M. Schmidt, D. Hara, J. D. Vega, M. B. Abuhaija, W. Tao, N. Dogan, A. Pollack, J. C. Ford, J. Shi, *Pharmaceutics* **2022**, *14*, 2205.

[15] D. B. Guerra, E. M. N. Oliveira, A. R. Sonntag, P. Sbaraine, A. P. Fay, F. B. Morrone, R. M. Papaléo, *Sci Rep* **2022**, *12*, 9602.

[16] K. Zhao, W. Ke, W. Yin, J. Li, M. Qiang, Z. Ge, *ACS Macro Lett* **2017**, *6*, 556.

[17] D. Li, J. Zhao, J. Ma, H. Yang, X. Zhang, Y. Cao, P. Liu, *Colloids Surf B Biointerfaces* **2022**, *211*, 112330.

[18] S. S. Mehrnia, B. Hashemi, S. J. Mowla, M. Nikkhah, A. Arbabi, *Radiat Oncol* **2021**, *16*, 33.

[19] M. Anuje, P. Pawaskar, A. Sivan, C. Lokhande, I. Ahmed, D. Patil, *J Med Phys* **2021**, *46*, 278.

[20] C. Wu, X. Du, B. Jia, C. Zhang, W. Li, T. C. Liu, Y. Q. Li, *J Mater Chem B* **2021**, *9*, 2314.

[21] Z. Liu, H. Tan, X. Zhang, F. Chen, Z. Zhou, X. Hu, S. Chang, P. Liu, H. Zhang, *Artif Cells Nanomed Biotechnol* **2018**, *46*, S922.

[22] S. J. Wu, J. Chen, B. Wu, Y. J. Wang, K. Y. Guo, *J Exp Clin Cancer Res* **2018**, *37*, 18.

[23] X. Hou, Y. X. Chang, Y. X. Yue, Z. H. Wang, F. Ding, Z. H. Li, H. B. Li, Y. Xu, X. Kong, F. Huang, D. S. Guo, J. Liu, *Adv Sci (Weinh)* **2022**, *9*, e2104349.
